# MASTL overexpression promotes chromosome instability and metastasis in breast cancer

**DOI:** 10.1038/s41388-018-0295-z

**Published:** 2018-05-10

**Authors:** Samuel Rogers, Rachael A. McCloy, Benjamin L. Parker, David Gallego-Ortega, Andrew M. K. Law, Venessa T. Chin, James R. W. Conway, Dirk Fey, Ewan K. A. Millar, Sandra O’Toole, Niantao Deng, Alexander Swarbrick, Paul D. Chastain, Anthony J. Cesare, Paul Timpson, C. Elizabeth Caldon, David R. Croucher, David E. James, D. Neil Watkins, Andrew Burgess

**Affiliations:** 1 The Kinghorn Cancer Centre, Garvan Institute of Medical Research, Darlinghurst, NSW 2010 Australia; 2 Children’s Medical Research Institute, The University of Sydney, Westmead, Australia; 3 Charles Perkins Centre, School of Life and Environmental Sciences, The University of Sydney, Sydney, NSW 2006 Australia; 4 Systems Biology Ireland, University College Dublin, Dublin 4, Ireland; 5 NSW Health Pathology, Department of Anatomical Pathology, St George Hospital, Kogarah, Sydney, New South Wales 2217 Australia; 6 School of Medical Sciences, UNSW Sydney, Kensington, NSW 2033 Australia; 7 School of Medicine and Health Sciences, Sydney Western University, Campbelltown, NSW 2560 Australia; 8 Sydney Medical School, Sydney University, Fisher Road, Camperdown, NSW 2006 Australia; 9 Department of Tissue, Pathology and Diagnostic Oncology, Royal Prince Alfred Hospital, Missenden Road, Camperdown, NSW 2050 Australia; 10 St. Vincent’s Clinical School, Faculty of Medicine, UNSW, Darlinghurst, NSW Australia; 11 College of Medicine at Rockford, Department of Health Sciences Education, University of Illinois, Rockford, IL 61107 USA; 12 School of Medicine and Medical Science, University College Dublin, Belfield, Dublin 4, Ireland; 13 School of Medicine, The University of Sydney, Sydney, NSW 2006 Australia; 14 Department of Thoracic Medicine, St Vincent’s Hospital, Darlinghurst, NSW 2010 Australia; 15 ANZAC Research Institute, University of Sydney, Sydney, NSW 2139 Australia

## Abstract

MASTL kinase is essential for correct progression through mitosis, with loss of MASTL causing chromosome segregation errors, mitotic collapse and failure of cytokinesis. However, in cancer MASTL is most commonly amplified and overexpressed. This correlates with increased chromosome instability in breast cancer and poor patient survival in breast, ovarian and lung cancer. Global phosphoproteomic analysis of immortalised breast MCF10A cells engineered to overexpressed MASTL revealed disruption to desmosomes, actin cytoskeleton, PI3K/AKT/mTOR and p38 stress kinase signalling pathways. Notably, these pathways were also disrupted in patient samples that overexpress MASTL. In MCF10A cells, these alterations corresponded with a loss of contact inhibition and partial epithelial–mesenchymal transition, which disrupted migration and allowed cells to proliferate uncontrollably in 3D culture. Furthermore, MASTL overexpression increased aberrant mitotic divisions resulting in increased micronuclei formation. Mathematical modelling indicated that this delay was due to continued inhibition of PP2A-B55, which delayed timely mitotic exit. This corresponded with an increase in DNA damage and delayed transit through interphase. There were no significant alterations to replication kinetics upon MASTL overexpression, however, inhibition of p38 kinase rescued the interphase delay, suggesting the delay was a G2 DNA damage checkpoint response. Importantly, knockdown of MASTL, reduced cell proliferation, prevented invasion and metastasis of MDA-MB-231 breast cancer cells both in vitro and in vivo, indicating the potential of future therapies that target MASTL. Taken together, these results suggest that MASTL overexpression contributes to chromosome instability and metastasis, thereby decreasing breast cancer patient survival.

## Introduction

In 2004, Greatwall kinase (Gwl) was identified as a novel and critical regulator of mitosis in *Drosophila* [1]. In 2009, this function was expanded to include the inhibition of the phosphatase PP2A-B55 [2], which was later shown to occur through the phosphorylation of α-endosulfine (ENSA) and the highly related protein Arpp19 [3, 4]. In 2010, MASTL (microtubule associated serine/threonine-like kinase), was identified as the human orthologue of Gwl, and was shown to be essential for inhibiting PP2A-B55 to permit timely entry into and progression through mitosis [5, 6]. Failure to inhibit PP2A-B55 caused premature dephosphorylation of mitotic substrates, and defects during mitotic exit, including chromosome segregation errors, cytokinesis failure and polyploidy. Similarly, complete knockout of MASTL in mouse embryonic fibroblasts causes mitotic collapse shortly after nuclear envelope breakdown (NEBD) [7], and premature silencing of the spindle assembly checkpoint [8]. Taken together, these data have established MASTL as a master regulator of phosphorylation during mitosis [9].

Although the role of MASTL in regulating mitosis is now well established, its roles in human biology and pathology are still poorly understood. However, several recent studies suggest that MASTL may play several critical roles in cancer biology, including stimulating oncogenic AKT kinase activity [10], regulating normal DNA replication timing [11] and recovery from pre-mitotic DNA damage checkpoint arrest [12]. MASTL is commonly overexpressed in several cancer types including colon, oral and breast cancer [10], with overexpression in oral and breast associated with cancer progression [13]. Notably, knockdown of MASTL can re-sensitise recurrent head and neck tumours to chemotherapy [13], and non-small cell lung cancer cells to radiation and chemotherapy [14]. Based on these data, we aimed to further examine the underlying mechanisms of how MASTL overexpression promotes oncogenesis. Here we present results showing high MASTL expression correlates significantly with chromosome instability and poor overall survival in patients with breast cancer. Overexpression of MASTL in immortalised normal breast epithelium cells delays cell cycle progression, drives aberrant cell division, disrupts migration, the actin cytoskeleton and cell–cell junctions leading to increased invasion and metastasis in vitro and in vivo. Taken together, these results indicate that MASTL is a novel breast cancer oncogene capable of over-coming contact inhibition, invasion and chromosome instability (CIN).

## Results

### MASTL overexpression correlates with poor patient outcomes in breast cancer

**Fig. 1:**
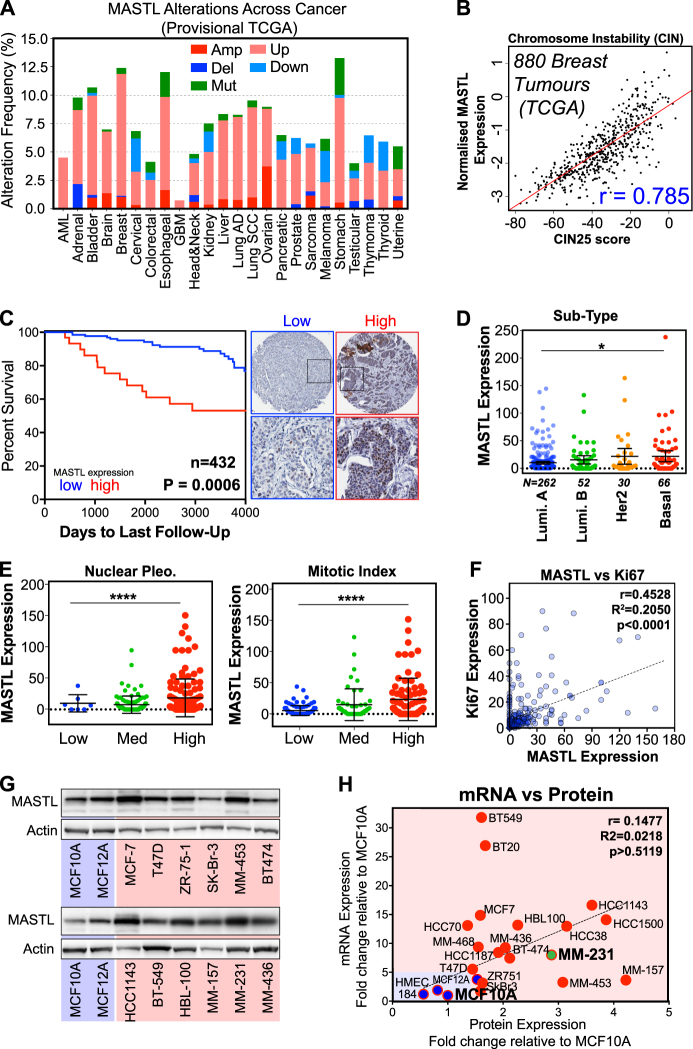
High MASTL expression correlates with poor overall survival in breast cancer. **a** The percentage of genomic alterations of MASTL across the spectrum of human cancers currently annotated in the TCGA provisional databases (cBioportal). **b** Correlation (Pearson) between the expression of CIN25 gene signature and MASTL in 880 breast tumour cases in the TCGA. **c** Breast cancer tumours from a cohort of 473 patients were stained and scored for MASTL protein expression and overall survival, Kaplan–Meier curves for high and low MASTL expression generated. Representative micrographs of low and high MASTL shown. **d**, **e** MASTL expression compared with other clinicopathological features; MASTL expression is significantly higher with mitotic index, histological grade and the Basal breast cancer subtype (one-way ANOVA, mean ± S.D, *n* = 473, ****p < 0.0001, *p < 0.05). **f** Correlation between MASTL expression and Ki67 staining intensity in TMA cohort (Pearson’s *r* correlation, 0.4522, *n* = 432). **g** Western blot analysis of MASTL protein expression in a range of non-tumorigenic (blue) and tumorigenic breast cancer cell lines (red). **h** Comparison of MASTL protein mRNA expression (TaqMan RT-PCR) from normal and breast cancer cell lines. All values normalised to MCF10A protein and mRNA levels

Previous reports have indicated that MASTL is overexpressed in several cancer types [10, 13], with overexpression in breast cancer correlating with poor patient outcomes [13, 15–17]. To analyse this further, we interrogated the publicly available provisional TCGA datasets for all major cancer types. The mutation rates of MASTL range from 0 to 4.8% across various cancer types, are spread across the length of the protein, with a potential truncating hotspot at K391 and a possible hyper-activating mutation at K72R (Figure S1A). The rates of amplification and deletion range from 0 to 2% (Fig. 1a). Overexpression of MASTL mRNA was observed in up to 10% of some cancer types. In breast cancer, MASTL is rarely mutated (0.5%), with overexpression and amplification more commonly observed (~10%). Given MASTLs well-reported role in regulating mitosis, we analysed the expression of MASTL in combination with CIN25 expression, a measure of chromosome instability, and found a strong positive correlation (*r* = 0.785), in the TCGA breast cohort (Fig. 1b). Using the online Kaplan–Meier plotter tool (KMPlot) [18], we performed univariate analysis of MASTL expression on 1764 breast cancer patients. Notably, increased MASTL expression significantly correlated with reduced distant metastasis-free survival (DMFS), post-progression survival (PPS) and relapse-free survival (RFS) (Figure S1B). Interestingly, overexpression of MASTL also correlated with multiple measures of poor patient outcome in lung and ovarian cancer (Figure S1C). Finally, MASTL protein overexpression in breast tumours was analysed in the Clinical Proteomic Tumour Analysis Consortium (CPTAC) and found to be highly significant for both reduced overall survival and poor progression free survival (Figure S1D).

To confirm these results, we analysed by tissue microarray (TMA) immunohistochemistry, 432 breast cancer patients from the St. Vincent’s Campus Outcome Cohort [19]. Univariate analysis of MASTL expression showed that high expression significantly correlated with poor overall survival (Fig. 1c). A small but significant enrichment for increased MASTL expression was seen in the basal compared with luminal A breast cancer subtype (Fig. 1d). A similar enrichment of high MASTL expression was also observed in the METABRIC TCGA breast cancer cohort [20], with ~44% of basal cases overexpressing MASTL (Figure S1E). In our patient cohort, elevated MASTL expression correlated significantly with higher mitotic index, nuclear pleomorphism and histological grade (Fig. 1e). In addition, high MASTL expression was also significantly correlated with increased Ki67 staining, suggested that MASTL may identify highly proliferative tumour cells (Fig. 1f). Next, we analysed MASTL protein and mRNA expression in a panel of 20 breast cancer cell lines (Fig. 1g and Figure S1F). A weak, positive correlation (*r* = 0.1477) between mRNA expression and MASTL protein levels was observed. Importantly, both MASTL mRNA and protein were overexpressed in breast cancer cells (red) compared with primary human mammary epithelial (HMEC, 184) and immortalised non-tumorigenic MCF10A and 12 A cells (blue, Fig. 1h). Taken together, these results suggest that high MASTL expression correlates with increased proliferation, tumour grade and CIN, and may be an independent prognostic factor for metastasis-free survival in breast cancer. These data are in agreement with several recent publications [15–17].

### Overexpression of MASTL delays cell proliferation and increases chromatin bridges

**Fig. 2:**
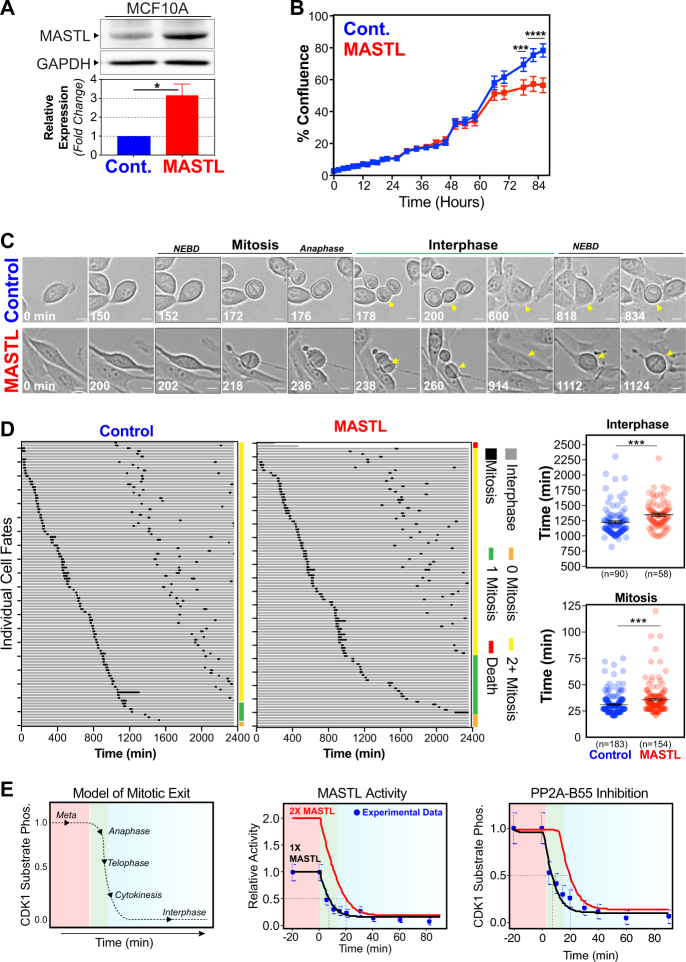
MASTL causes mitotic defects by delaying cell cycle progression. **a** MCF10A cells transfected with lentivirus coding for MASTL (MASTL) or empty vector controls (Cont.) were analysed by western blot, for MASTL and GAPDH (loading control). MASTL protein overexpression was determined by densitometry (mean ± SEM, *n* = 3, unpaired Student's *t*-test, *p < 0.05). **b** Cell proliferation was assessed using an IncuCyte Zoom over 72 h. Proliferation was measured by %Confluence of the phase contrast channel (mean ± SEM, *n* = 3, two-way ANOVA with multiple corrections, ***p < 0.001, ****p < 0.0001). **c** Live-cell microscopy of control and MASTL cells. **d** Individual cell fate maps for 100+ cells. Average interphase (from first anaphase to second NEBD), and mitotic length (NEBD to anaphase), are shown (mean ± SEM, unpaired Student's *t*-test, ***p < 0.001). **e** Mathematical model of mitosis (adapted from Rogers et al. [21]), incorporating twofold increase in MASTL activity to mimic the effects of MASTL overexpression on PP2A-B55 reactivation during mitotic exit

The above results show that MASTL correlates with increased proliferation and CIN in patient tumours. To determine if MASTL overexpression directly drives these phenotypes, we stably transduced the immortalised non-tumorigenic breast cell line MCF10A, with a bicistronic lentiviral construct that co-expressed mCherry fluorescent protein and either full-length MASTL or an empty vector (Control). Cells were transduced, selected and sorted by flow cytometry using mCherry, to ensure homogeneous expression levels of the lentiviral constructs. The overexpressing cell lines produced approximately threefold more MASTL protein compared with the controls (Fig. 2a). MASTL overexpression slightly delayed monolayer cells reaching confluence compared with control cells (Fig. 2b), suggesting that MASTL overexpression was slowing cell proliferation. To analyse this delay in greater detail, we performed time-lapse microscopy on asynchronously growing cells coupled with single-cell tracking (Fig. 2c). Over 90% of control cells completed two cell divisions within the 48-h timeframe, with an average interphase length (from first anaphase to second nuclear envelope breakdown) of 1225 min, and mitotic length (NEBD to anaphase) of 31.1 min (Fig. 2d). In contrast, MASTL-overexpressing cells were less likely to successfully complete two divisions within the timeframe, with ~25% of cells either dying, completing only one or no cell divisions. In addition, cells that completed two division took significantly longer, with an average interphase length of 1349 min. Furthermore, MASTL-overexpressing cells also took slightly longer (~5 min) to complete mitosis with an average length of 35.8 min. These increases were associated with a doubling of mitotic defects arising from increases in aberrant cleavage and multipolar mitosis events (Figure S2A). Quantitative immunofluorescence analysis of DNA content showed that MASTL overexpression caused a slight shift towards increased nuclear size compared with control cell, and a significant increase in the rate of micronuclei from ~4.5 to ~8% (Figure S2B). This correlated with a significant increase in the presence of anaphase chromosome bridges (Figure S2C). To understand how increased MASTL expression could be delaying mitosis, we utilised our previously developed mathematical model of mitotic exit [21]. Using this model, we simulated a doubling of MASTL activity to mimic a twofold increase in MASTL expression (Fig. 2e). Interestingly, this caused a slight delay in reactivation of PP2A-B55 preventing the timely dephosphorylation of CDK1 substrates and shifting the onset of mitotic exit by ~10 min. This is consistent with our above results and previous reports that show knockdown of PP2A-B55 delays mitotic exit by ~10 to 15 min in human cells [22]. Interestingly, knockdown of PP2A-B55 disrupted mitotic exit events including correct formation of the central spindle, nuclear envelope and cleavage furrow [23]. Taken together, these data indicate that MASTL overexpression could delay and disrupt the normal timing of mitotic exit, potentially explaining the observed increase in chromosome bridges and micronuclei.

### Quantitative phosphoproteomic analysis of MASTL-overexpressing cells identifies novel signalling pathways independent of AKT hyper-activation

**Fig. 3:**
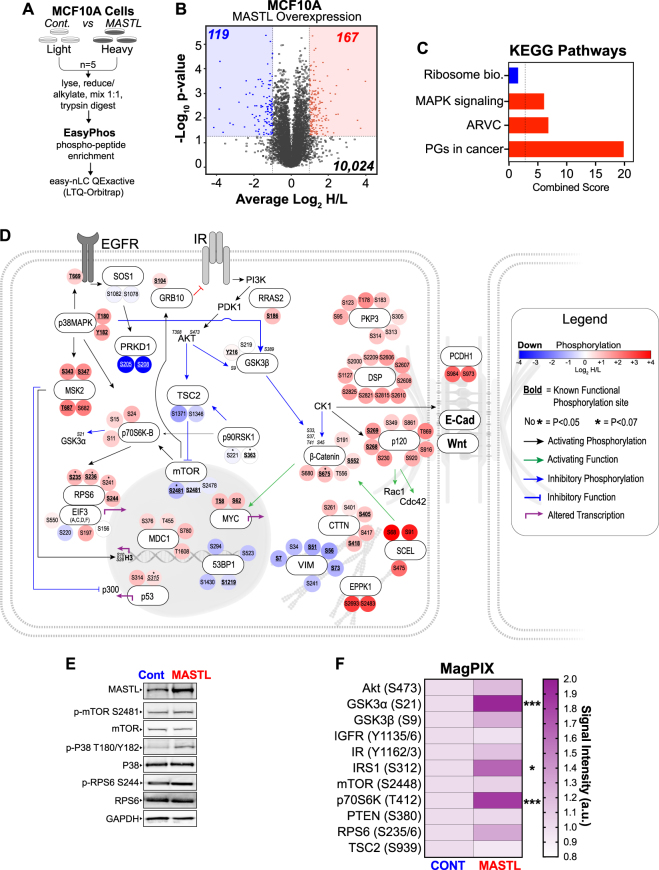
MASTL overexpression increases the phosphorylation of proteins involved in actin, stress kinase signalling pathways. **a** Experimental design, and SILAC workflow. MCF10A control and MASTL were grown for >6 doubling in heavy and light SILAC media. Heavy and light cultures were harvested reduced and alkylated, mixed 1:1, acetone precipitated and then digested with LysC/Trypsin. Phosphopeptides were enriched using the “EasyPhos” method, enriched peptides were then analysed on easy-nLC1200 coupled to a QExactive-HF mass spectrometer. **b** Volcano plot depicting the average Log_2_H/L ratio and –Log_10_P-value of the detected phosphopeptides. These were grouped into two populations according to significance (*p* < 0.05 cut-off, y~1.2 dotted line, *n* = 1961) and either; Log_2_H/L < −1 (DOWN; blue shading, x = −1 dotted line, *n* = 119), or Log_2_H/L > 1 (UP; red shading, x = 1 dotted line, *n* = 167). **c** KEGG pathway analysis of top UP and DOWN phosphosites. **d** Manual curation of top significant UP and DOWN phosphosites into an interconnected signalling network. The interaction between each protein is denoted by the arrow connecting the proteins. Sites that were close to significant indicated with * (*p* < 0.07). Specific phosphosites are colour-coded from blue (DOWN) to red (UP), based on observed Log_2_ ratios. Known functional phosphosites annotated in the PhosphoSitePlus database underlined in bold. **e** Western blot validation of the mass spectrometry results. MASTL and control lysates probed with total and phospho-specific antibodies (blots are representative of three biological replicates). **f** Heatmap from MagPix assay showing fold-change in phosphorylation of key proteins involved in AKT signalling between Control and MASTL cell lysates. Overexpressing cells normalised to the background signal, and relative to control (*n* = 3, unpaired *t*-test, *p < 0.05, ***p < 0.001)

Several recent reports have shown that MASTL may have several additional roles outside of mitosis, including regulation of DNA replication [11], recovery from DNA damage [24] and hyper-activation of AKT signalling [10]. Therefore, to better understand how MASTL overexpression could be driving breast cancer, we undertook an unbiased phosphoproteomic screen using stable isotope labelling of amino acids in cell culture (SILAC) (Fig. 3a). Asynchronous MCF10A control and MASTL-expressing cells were grown in light or heavy (Lys-8, Arg-10) amino acids, as previously described [21, 25]. We identified 10,024 phosphopeptides across the five replicates, with good correlation between each run (Figure S3A). Using a twofold cut-off (Log_2_H/L ratio < −1 or > 1) and a *p*-value < 0.05, we identified 167 candidate phosphopeptides (on 91 proteins) that were significantly increased (UP, red), and 119 (from 63 proteins) that were decreased (DOWN, blue) upon MASTL overexpression (Fig. 3b and Table S1). Phosphorylation of the MASTL substrate ENSA on S67 was consistently increased in the MASTL-overexpressing cells (*p* = 0.02), however, the increase (~0.4) was below our significance threshold of 1 (Table S1). Notably, there was no overlap between the proteins present in the UP and DOWN groups (Figure S3B). ClueGO enrichment analysis identified strong enrichment for proteins involved in hemidesmosome assembly, cell–cell junctions and gene transcription in the UP phosphopeptides. Similarly, KEGG pathway analysis identified desmosome dysfunction (arrhythmogenic right ventricular cardiomyopathy), mitogen-activated protein kinase (MAPK) signalling, and proteoglycans in cancer as significantly enriched in the UP phosphopeptides following MASTL overexpression (Fig. 3c and Figure S3C). Potential upstream kinases for significant phosphopeptides were annotated using GPS 3.0 [26], with notable increases in casein kinase 1 (CK1) and decreases in cyclin dependent kinase (CDK) signalling observed (Figure S3D). To further examine this, we generated a network map of the top UP and DOWN phosphorylated proteins (Fig. 3d). As predicted by ClueGO and KEGG analysis, UP phosphosites were clustered around desmosomes (e.g., DSP, p120, PCDH1 and B-catenin), actin dynamics (SCEL and EPPK1), MAPK signalling (p38, MSK2, p70S6K and RPS6) and DNA damage signalling (p53, MDC1 and 53BP1). In contrast, DOWN sites were clustered around protein kinase D1 (PRKD1), vimentin and downstream AKT targets (TSC2 and mTOR), although AKT itself was not detected. Using currently available phospho-specific antibodies, we confirmed that phosphorylation on T180/Y182 of p38MAPK was increased (~twofold), whereas a much smaller increase was seen for RPS6 (S244) and mTOR (Ser2481) remained unchanged (Fig. 3e), consistent with the mass spectrometry results. To assess PI3K/AKT/mTOR (Phosphatidylinositol-4,5-bisphosphate 3-kinase/Protein Kinase B/mammalian target of rapamycin) pathway activity, we next utilised an antibody-based multiplex (MagPix) phosphoprotein screen. A significant increase in the activating phosphorylation site of p70S6K (T412), and of its downstream substrates IRS1 (S312), GSK3α (S21) and RPS6 (S235/6) was observed upon MASTL overexpression (Fig. 3f). A small increase in AKT (S473) and its substrate GSK3β and was observed, but these were not significant. Although pathway members mTOR (S2448), phosphatase and tensin homolog (PTEN, S380) and TSC2 (S939) remained unchanged. Analysis of total and phosphoprotein data from the TCGA provisional breast cancer cohort, similarly identified disruption to PI3K/AKT/mTOR signalling, p38/MAPK (Toll-like signalling) and p53 DNA damage checkpoint responses (Figure S4A and S4B). Interestingly, MASTL overexpression was also strongly associated with mutation of p53 (Figure S4B, inset). Additional analysis of our breast TMA cohort found a significant positive correlation between MASTL protein expression and p-AKT (S473), cyclin B, cyclin A, p16 and p53 levels (Figure S4C). We confirmed these results in a panel of breast cancer cell lines and found positive correlations between MASTL and p-AKT (S473), total AKT, cyclin B1, p53 and p-p38 levels, although p-AKT and total p38 were just above the <0.05 significance threshold (Figure S4D). Taken together, these results suggest that MASTL overexpression correlates with deregulation of desmosomes, actin regulatory proteins, disruption to PI3K/AKT/mTOR signalling and an increase in DNA damage signalling across patient tumour samples, breast cancer cell lines and our MCF10A model system.

### MASTL overexpression disrupts cell migration and contact inhibition

**Fig. 4:**
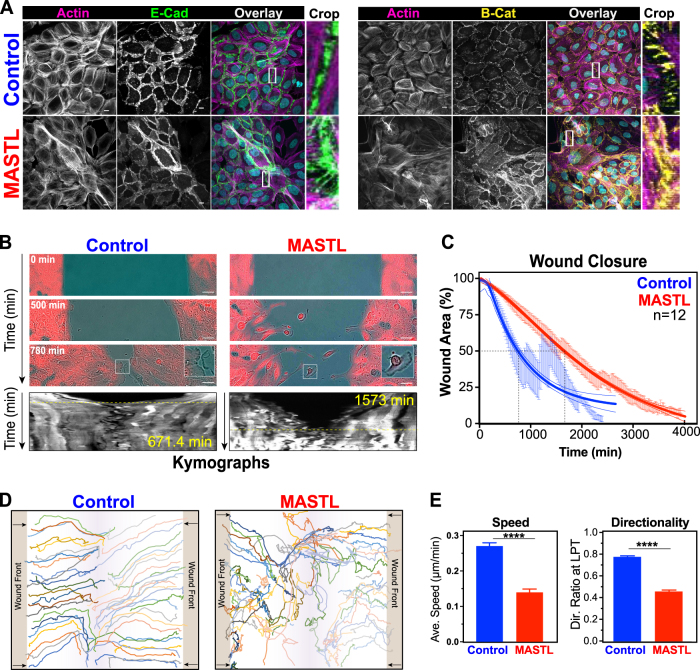
MASTL overexpression results in the loss of cell–cell junctions, causing migration defects. **a** Representative maximum projection images from confocal immunofluorescence of control and MASTL stained with H33342 (cyan) for E-Cadherin (green), β-catenin (yellow), and phalloidin (F-actin, pink). Scale bar 10 µm. **b** Wound-healing assays of control, and MASTL cell lines. Representative phase contrast, with inset of invading cell front (Control) and individual cells (MASTL) shown. Scale bar 50 µm. Kymographs (mCherry) generated by horizontal line through middle of wound area. Yellow dotted line indicates wound closure. **c** MRI Wound-Healing Tool for ImageJ was used to determine unclosed wound area as a function of time (mean ± SEM shown). Dotted line indicates 50% closure. Trend-line analysis performed using asymmetric sigmoidal analysis in Prism (*R*^2^ = 0.5888; Control and 0.7912; MASTL). **d** The MTrackJ plugin for ImageJ was used to track individual cells (*n* = minimum 50 cell/condition) from **b**. **e** Data from **d** were analysed using the DiPer software tool. Shown are average speed and directionality ratios for MASTL and controls from three independent experiments (mean ± SEM, unpaired *t*-test, ****p < 0.0001)

Loss of contact inhibition is a hallmark of cancer and is commonly associated with disruption of PI3K/AKT/mTOR signalling and desmosomes. Focus formation assays showed that MCF10A control cells formed a uniformly stained single monolayer, in contrast, MASTL overexpression allowed cells to grow on top of each other creating a mesh-like network (Figure S5A), consistent with a loss of contact inhibition, as previously reported [10]. Confocal immunofluorescence analysis confirmed that control cells formed a typical ‘cobblestone’ morphology at confluence with clear cell–cell junctions and a well-organised actin fibre network (Fig. 4a). In contrast, MASTL-overexpressing cells failed to form a single monolayer, with cells growing on top of each other. This correlated with a disorganisation of the actin cytoskeleton, E-cadherin and β-catenin localisation (Fig. 4a and Figure S5B), and slight reduction in expression of E-cadherin and β-catenin protein levels (Figure S5C) suggesting cells have poorly defined cell–cell junctions. The elongation of cells, loss of contract inhibition, disruption of actin cytoskeleton and cell–cell junctions suggested that MASTL overexpression may be causing an epithelial-to-mesenchymal transition (EMT). Notably, mesenchymal cells gain migratory and invasive properties, therefore, migration defects were assessed using a two-dimensional (2D) wound-healing assay (Fig. 4b). Control cells migrated into the wound as a collective monolayer. In contrast, MASTL overexpression disrupted the collective migration with an increased number of cells migrating individually. This corresponded with a significant delay in the time required to close the wound for MASTL-overexpressing cells (Fig. 4c). Single-cell tracking (Fig. 4d) confirmed that control cells rapidly migrated in a highly directional manner into the wound area. In contrast, MASTL cells appeared to randomly migrate around the wound, resulting in a significant reduction in directionality, and an overall slower average migration speed (Fig. 4e). Taken together, these data indicate that MASTL overexpression disrupts normal cell–cell junctions and actin organisation resulting in a loss of contact inhibition and partial EMT.

### MASTL overexpression promotes increased cell growth and proliferation in 3D

**Fig. 5:**
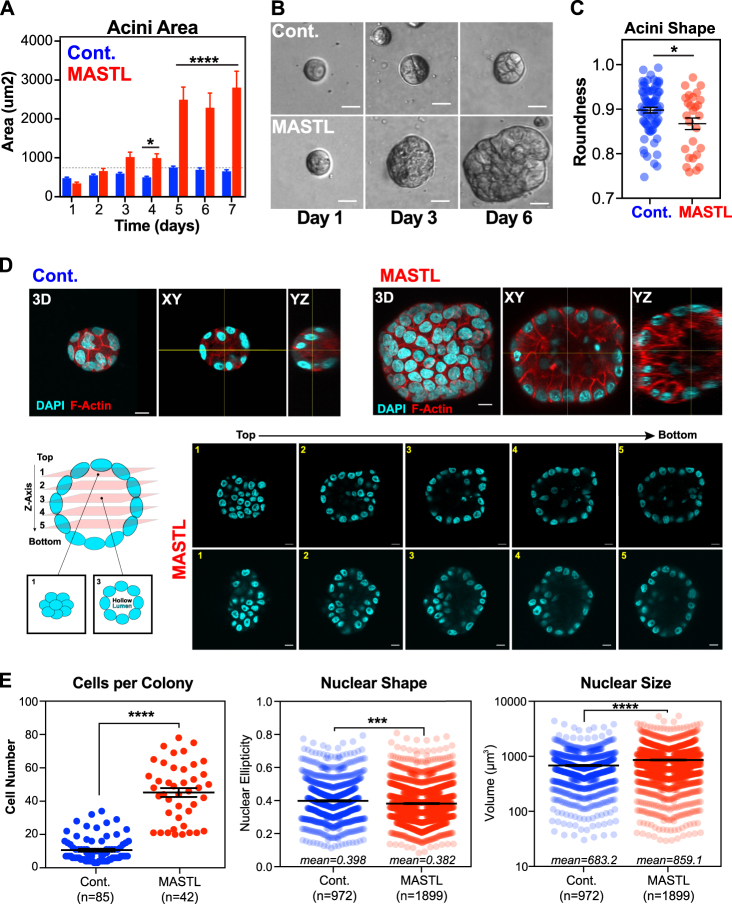
MASTL overexpression increases proliferation and impairs organoid architecture in 3D culture. Cell lines were seeded into matrigel (Corning) and cultured under reduced serum and EGF conditions. **a-c** Phase contrast photos taken every day for 7 days and 20–40 individual colonies for each condition and time-point were measured in ImageJ for area and roundness. Shown are mean ± SEM from three independent biological replicates. For **a**, two-way ANOVA with multiple corrections, *p < 0.05, ****p < 0.0001. For **c**, unpaired *t*-test, *p < 0.05. **d** Seven-day acini were fixed and stained with H33342 (cyan) and phalloidin (red) and analysed by confocal microscopy with 0.3 µm z-stacks from the top of the acini to bottom shown, along with 3D projections and XY/YZ cross-sections to identify cleared lumens. **e** 3D acini from **d** were analysed using Imaris and individual nuclei were counted and measured for shape (ellipticity) and size (unpaired *t*-test, ***p < 0.001, ****p < 0.0001)

In tumours, MASTL overexpression correlates with increased proliferation markers (Figs. 1e, f), however, in 2D cell culture we found that MASTL overexpression delayed interphase transit time and reduced cell proliferation (Figs. 2b-d). To better understand the role of MASTL in breast cancer, we utilised 3D spheroid culture [27], which better represents the tumour microenvironment [28]. In 3D culture, MCF10A cells undergo an initial growth phase over 4–5 days and form spheroids that become contact inhibited. This triggers apoptosis of the internal cells, creating a hollowed lumen by day 7 [27]. Control cells followed this pattern with growth peaking at day 5, and small well-rounded acini formed by day 7 (Figs. 5a-c). MASTL-overexpressing acini grew at a similar rate over the first 3 days, but by day 4, they rapidly expanded and were ~4.3-fold larger in size and misshapen (Figs. 5a-c). A failure to initiate apoptosis could explain the increased acini size, therefore, to determine if acini were correctly forming hollow lumens, 3D cultures were analysed by confocal immunofluorescent microscopy (Fig. 5d). As expected, the luminal space in controls was void of internal cells. Similarly, the larger MASTL acini were mostly void of cells (Fig. 5d), with the misshapen appearance likely due to invagination of the outer cells into the empty luminal space. This suggests that MASTL overexpression does not prevent the normal apoptotic-induced clearing of luminal cells. In contrast, MASTL overexpression did result in a ~3.7-fold increase in the average number of cells (Fig. 5e), indicating that the increased acini size was caused by continued cell proliferation. In support, MASTL acini often contained nuclei with condensed and aligned chromosomes indicative of mitosis (Figure S5D, M). In addition, there was a slight decrease in nuclear eccentricity and an increase in nuclear size in MASTL-overexpressing acini (Fig. 5e), correlating with the increased nuclear size observed in 2D (Figure S2B), and nuclear pleomorphism in patient TMAs (Fig. 1e).

### MASTL overexpression induces DNA damage and replication stress

**Fig. 6:**
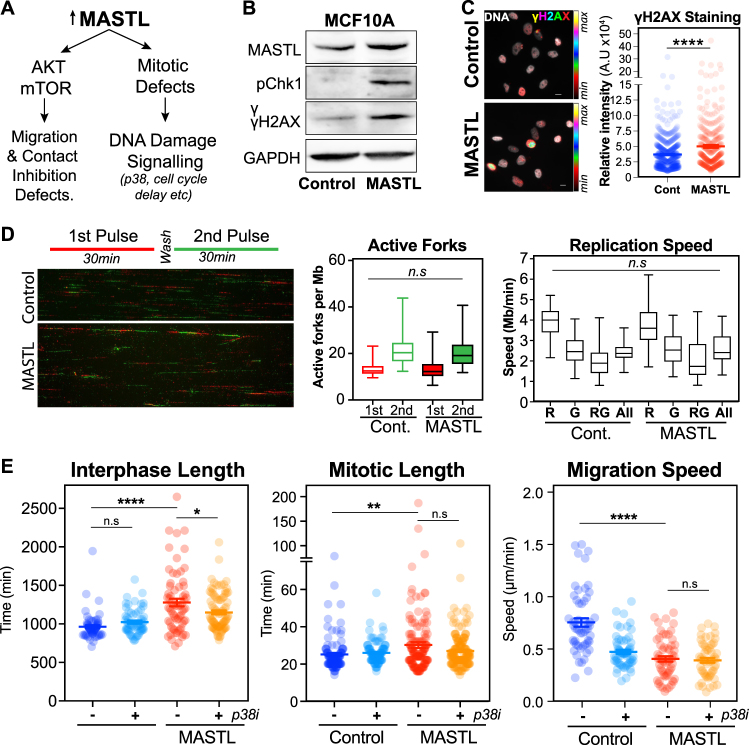
MASTL overexpression induces a G2 DNA damage checkpoint response. **a** Schematic of two major pathways disrupted by MASTL overexpression. **b** Western blot analysis of asynchronous MCF10A cells. **c** Immunofluorescence analysis of cells (minimum *n* = 1000) from **b**. Scale bar 10 µm. **d** DNA fibre analysis of cells from **b.** A minimum of 200 fibres were analysed using computer aided scoring and analysis (CASA) software. **e** Live-cell microscopy of the control and MASTL cells treated with or without 10 µm of SB203580 (p38i). Average interphase (from first anaphase to second NEBD), and mitotic length (NEBD to anaphase), are shown, along with migration analysis using MTrackJ and DiPer. Shown are mean ± SEM (one-way ANOVA Student's *t*-test, *p < 0.05, **p < 0.01, ***p < 0.001, ****p < 0.0001) for *n* = 50 and 100 cell for Control ± p38i and MASTL ± p38i, respectively

The other major alteration we observed was an increase in DNA damage checkpoint signalling upon MASTL overexpression, suggesting that there was significant DNA damage. In support, we observed a delay in interphase and mitotic progression, increased nuclear size and increased chromosome segregation errors (Fig. 2d and S2B,C). Western blot and immunofluorescence analysis of asynchronous MCF10A cells showed a clear increase in both pChk1 and γH2AX (Figs. 6b, c), consistent with activation of interphase DNA damage checkpoints. Interestingly, DNA fibre analysis found no significant difference in the number of active origins or replication speed upon MASTL overexpression (Fig. 6d). This suggests that the delay most likely occurs during G2, which correlates with the observed increase in nuclear size (Figure S2B) and the increase in p38 phosphorylation, which is a known regulator the G2/M DNA damage checkpoint response [29, 30]. To confirm this, we treated asynchronous MCF10A cells with the p38 inhibitor SB203580 and analysed cell cycle transit by live cell imaging. Importantly, inhibition of p38 rescued the MASTL-induced interphase and mitotic delays, increasing the number of cells completing two or more divisions within 48 h (Fig. 6e, Figure S6A, B). Notably, p38 activity has also been shown to regulate EMT in MCF10A cells [31, 32]. However, although inhibition did reduce migration in controls, no significant effect was observed in MASTL overexpression cells (Fig. 6e and S6C). Taken together, these data indicate that the observed increase in p38 signalling upon MASTL overexpression is a G2 checkpoint-specific response.

### Knockdown of MASTL prevents invasion and migration in breast cancer cells

**Fig. 7:**
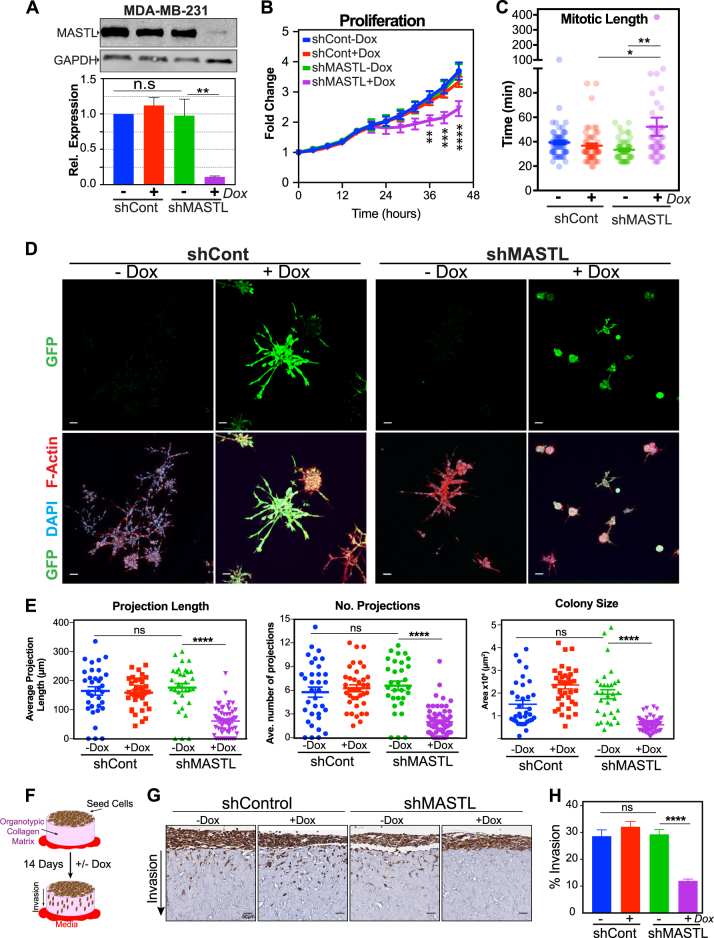
Knockdown of MASTL severely impairs the proliferative, and migratory capacity of cancerous cell lines. **a** Western blot analysis of MDA-MB-231 cells stably expressing doxycycline inducible bicistronic vectors containing GFP and either shMASTL or scramble controls (shCont). Cells were treated for 72 h with either vehicle, or 1 µg/mL doxycycline (+DOX). Representative blots and quantification from three independent experiments shown (mean ± SEM, *n* = 3, two-way ANOVA multiple comparisons, n.s = not significant; **p < 0.01). **b** IncuCyte Cell proliferation assay. Total cell number (nuclei measured by H2B-mCherry), expressed relative to starting time (mean ± SEM shown, *n* = 3). Two-way ANOVA with multiple comparisons. **c** Timelapse single cell analysis was used to quantify average mitotic length (NEBD to anaphase). Mean ± SEM is shown, *p < 0.05, **p < 0.01. **d** Cells were seeded into matrigel and maintained in complete media ± DOX for 7 days. Colonies were fixed and stained for DNA (DAPI, cyan), filamentous actin, F-actin (Phalloidin, red), and shRNA expression was checked by GFP (green) expression. Scale bar 50 µm. **e** The length of stellate projections, number of projections and average central colony size were quantified from a minimum of 30 colonies from three biological replicates (n.s. not significant, two-way ANOVA, ****p < 0.0001). **f** Schematic representation of Organotypic invasion assay, where MDA-MB-231 cells are seeded on top of a organotypic collagen matrix and allowed to invade for 14 days toward a chemotactic media gradient. **g** Representative images of the α-cytokeratin IHC stained organotypic collagen matrix, MDA-MB-231 cells stained brown. Scale bar 50 µm. **h** Quantification of 30 random fields from three independent experiments for each condition were scored. Shown are the percentage of the cells relative that invaded into the collagen relative to the cells that failed to invade (mean ± SEM, two-way ANOVA with multiple comparisons, ns = not significant, ****p < 0.0001)

The above results indicate that overexpression of MASTL prevents contact inhibition and increases mitotic errors leading to DNA damage. These effects result in increased cell growth, proliferation and invasion in 3D, potentially explaining the correlation with CIN and poor patient survival. To further validate this, we next determined the dependence of malignant breast cancer cells on MASTL overexpression. MDA-MB-231 cells were chosen as they are a well-established highly invasive breast cancer cell line, which overexpress MASTL approximately threefold compared with MCF10A cells (Figs. 1g, h). To induce knockdown of MASTL, MDA-MB-231 cells expressing H2B-mCherry were stably transduced using an inducible lentiviral vector containing a bicistronic internal ribosome entry site (IRES) vector with green fluorescent protein (GFP) and short hairpin RNA (shRNA) against MASTL (shMASTL) or a scramble control (shControl). Doxycycline (Dox) induction for 72 h resulted in ~70–90% knockdown for MASTL compared with shControl and uninduced cells (Fig. 7a). IncuCyte live cell proliferation assays identified a small, significant reduction in total cell numbers upon MASTL depletion (Fig. 7b). Single-cell tracking identified that in interphase transit time was not significantly altered upon depletion of MASTL (Figure S7A), however, there was a significant increase in mitotic transit time (Fig. 7c and S7B). This corresponded to an increase in aberrant divisions (Figure S7C, S7D), which resulted in a 20% increase in cells that completed one or less divisions during the time course (Figure S7B, S7E). These defects are similar to those previously reported by ours and other laboratories [6], supporting the specific knockdown of MASTL.

To further assess the effects of MASTL knockdown, we analysed cell growth in 3D cell culture. Both shControl and shMAST cells were grown in Matrigel for 7 days in the absence (–Dox) or presence (+Dox) of Dox. Addition of Dox resulted in efficient GFP production indicating shRNA expression (Fig. 7d). Both uninduced and Dox-treated shControl cells produced large acini with multiple projections extending on average 175 µm from the central mass (Fig. 7e). No significant difference was observed between uninduced shMASTL acini and shControl cells; however, knockdown of MASTL significantly reduced acini size, along with the number and length of projections (Figs. 7d, e). To assess invasion, we performed 3D organotypic invasion assays [33]. Briefly, 1 × 10^5^ MDA-MB-231 cells were seeded on fibroblast-contracted collagen matrices and allowed to grow for 4 days. 3D matrices were then moved to an air–liquid interface, which provides a chemo-attractive gradient for cells to invade over a 14-day period (Fig. 7f). Approximately 30% of shControl cells and uninduced shMASTL cells successfully invaded into the collagen matrix (Fig. 7g). In contrast, knockdown of MASTL significantly reduced invasion by ~20%, with ~90% of cells remaining on top of the matrix (Figs. 7g, h). Taken together, these results indicated that MASTL knockdown prevents invasion in the metastatic MDA-MB-231 breast cancer cell line.

### MASTL knockdown blocks invasion and metastasis in vivo

**Fig. 8:**
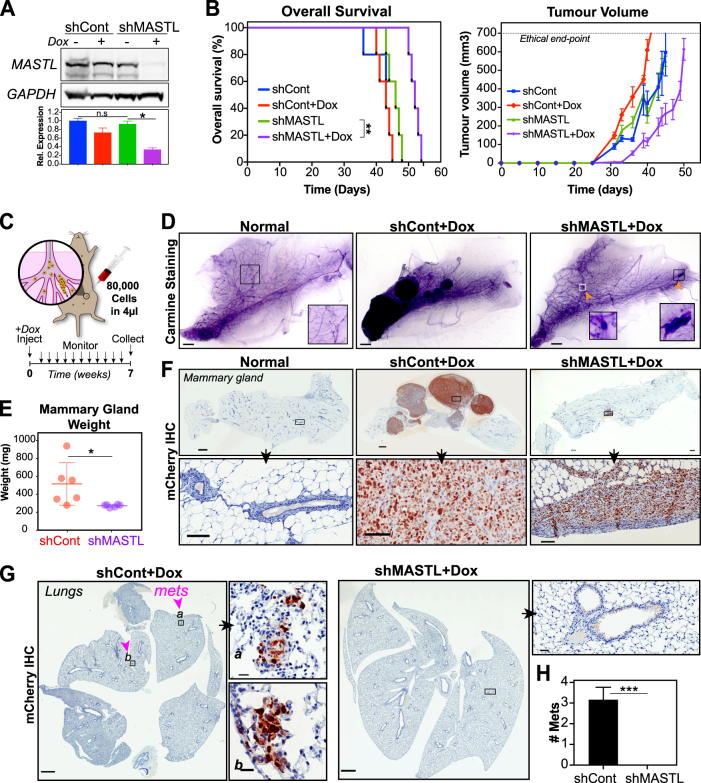
Knockdown of MASTL in vivo delays tumour growth, increases survival and blocks metastasis. **a** Similar to Fig. 6a, MDA-MB-231 cells stably expressing inducible shMASTL or scramble controls (shCont) were injected into mammary fat pads of NSG mice. Mice were fed DOX or control diet, and tumours were harvested at ethical endpoint. MASTL expression was analysed by western blot staining for anti-MASTL and GAPDH (loading control), and quantified by densitometry (mean ± SEM, *n* = 5, nonparametric Kruskal–Wallis test, ns = not significant, **p < 0.01). **b** Kaplan–Meier survival curve of time to ethical endpoint (*n* = 5, Log-rank Mantel-Cox test, **p = 0.0018) and tumour volume. **c** Schematic of intraductal xenograft model. Briefly, 80,000 MDA-MB-231 shControl or shMASTL cells were injected in 4 µl via nipple incision into mammary ducts. All mice were fed DOX diet, assessed twice weekly and all tissues were collected at 7 weeks. **d** Representative carmine stained mammary whole mounts from mice described in **c**, scale bars 1 mm. Normal mammary glands were not injected with any cells. **e** Mammary gland (including tumour) weight at endpoint of mice described in **c** (*n* = 6, Student's *t*-test, *p < 0.05). **f** Mammary glands from **d** were destained, rehydrated and formalin fixed. Immunohistochemical analysis of mCherry expression to identify tumour cells, scale bars 1 mm and 100 µm. **g** Immunohistochemically analysis of lungs collected from mice described in **c**. Lungs were inflated and formalin fixed, then stained with anti-mCherry to detect tumour cells. Scale bars 1 mm and 100 µm. **h** Quantification of total number of metastatic lesions found within lungs of individual mice from **g**. (*n* = 6, Student's *t*-test, ***p < 0.001)

Previous studies have shown that additional MASTL overexpression in MDA-MB-231 cells can promote primary tumour growth in mouse xenograft models [10]. However, the effects of MASTL knockdown on primary tumour growth vary from no effect in head and neck cancer [13] to delayed tumour growth in breast cancer [10]. Furthermore, the ability of MASTL to regulate metastasis in vivo has not yet been assessed. Therefore, we wanted to determine if MASTL knockdown could affect both primary tumour growth and metastasis in vivo. To assess the effects of MASTL knockdown on primary tumour growth, 500,000 MDA-MB-231 shControl or shMASTL cells were injected into the mammary fat pads of immune-compromised NOD Scid IL2 gamma–/– (NSG) mice. Mice were fed Dox (+Dox) or a control diet (–Dox). Tumours were monitored twice weekly and mice were culled when tumours reached ethical endpoint (10% tumour burden). Western blot analysis of the resected primary tumours confirmed that MASTL protein levels were significantly depleted upon Dox treatment in shMASTL cells (Fig. 8a). Importantly, knockdown of MASTL resulted in a significant delay in tumour initiation and reduced tumour size, which resulted in an increase in overall survival (Fig. 8b), supporting previous reports that MASTL knockdown reduces primary tumour growth [10].

To determine the effects of MASTL on invasion and metastasis, we utilised the mouse intraductal xenograft model (MIND), which better recapitulates the clinical progression of breast cancer from in situ carcinoma to metastatic disease [34]. In the MIND model, cancer cells are placed in the mammary tissue niche during tumour establishment and are required to breach the basement membrane of the mammary duct in order to become locally invasive and metastatic. Briefly, 80,000 shControl or shMASTL cells were injected into mouse mammary ducts via nipple incision (Fig. 8c). All mice were fed a Dox diet, monitored twice weekly and sacrificed at 7 weeks. Whole mammary glands (including primary tumour), were weighed and collected for Carmine staining and subsequent immunohistochemistry (IHC) analysis. Carmine staining of whole mammary glands clearly showed a well-developed duct system in normal mammary tissue, whereas shCont (shCont + Dox) mice contained multiple large growths that engulfed the majority of the mammary gland (Fig. 8d). In contrast, depletion of MASTL (shMASTL + Dox) reduced the size and number of growths (Fig. 8d), resulting in a significant reduction in total mammary gland weight (Fig. 8e). Immunohistochemistry staining of sections with anti-mCherry confirmed that the large growths in shControl mice were from the injected MDA-MD-231 cells (Fig. 8f). Similarly, shMASTL + Dox mice contained small tumours at the initial injection site (mCherry positive), which failed to migrate further into the mammary gland, confirming cells do embed into the mammary gland ducts, but fail to invade through the basement membrane. To assess metastasis, immunohistochemistry staining of lung tissue for mCherry was also performed (Fig. 8g). The lungs of shControl mice contained on average three small metastases, whereas no metastasis were found in any sections from shMASTL-depleted mice (Figs. 8g, h). Taken together, these results suggest that knockdown of MASTL can block both tumour growth and prevent the invasion and metastasis of cancer cells in vivo.

## Discussion

Here we provide additional evidence supporting that MASTL overexpression acts as a novel driver of breast cancer, capable of increasing CIN, invasion and metastasis. We believe that MASTL achieves this through two major pathways (Fig. 6a). The first is through disruption of cell junctions and the actin cytoskeleton, which results in a partial EMT and loss of contact inhibition, with MASTL cells becoming more elongated and migrating as disorganised individual cells. This corresponded with disruption of PI3K/AKT/mTOR signalling in our MCF10A model and correlated with increased activation of AKT (S473 phosphorylation) in patient tumour samples. This is consistent with a previous report showing that MASTL hyper-activates the AKT pathway [10], and likely explains how MASTL drives continued proliferated under normally repressive in vitro and in vivo environments.

Second, we identified a new role for MASTL overexpression directly disrupting and delaying the correct timing of mitotic exit. Mathematically modelling suggests that this delay is due to continued inhibition of PP2A-B55 phosphatase by MASTL phosphorylation of ENSA [3, 4, 35]. Notably, loss of PP2A-B55 [22, 23], reduced CDK1 activity [36], Arpp19 and cyclin B1 overexpression in yeast [37] have all been shown to increase the rate of erroneous chromosome segregation and cytokinesis defects resulting in increased micronuclei formation and CIN. Thereby, providing a logical explanation for the observed correlation between MASTL overexpression and increase in CIN in patient tumours. Notably, micronuclei are often reincorporated into daughter nuclei, which in turn can cause DNA damage and Chromothripsis [38, 39]. This likely explains why MASTL-overexpressing cells showed an increase in DNA damage (𝛾H2AX), Chk1 phosphorylation and p38 stress kinase signalling and took significantly longer to transit interphase. Cell cycle checkpoint activation of p38 commonly occurs in response to DNA damage [29, 40, 41], phosphorylating CDC25B and delaying cells in G2 [30]. In support, inhibition of p38 rescued the interphase delay supporting that delay was a G2 checkpoint response. Interestingly, functional p53 is also required for a prolonged and sustained G2 checkpoint responses following DNA damage and aberrant mitotic divisions [42]. Notably, MASTL-overexpressing tumours commonly contain mutated p53, a combination that would provide a significant growth advantage allowing the unrestricted and defective mitotic divisions necessary for ongoing CIN. Interestingly, CIN has recently been shown to be a driver of metastasis [43], whereas deregulation of the PI3K-AKT pathway can disrupt DNA replication and repair [44]. Consequently, future studies analysing the potential cross-talk between the two MASTL-driven mechanisms will be of great interest. Finally, we showed that knockdown of MASTL delayed primary tumour growth, and prevented invasion and metastasis of MDA-MB-231 cells, resulting in a significant increase in overall survival. The ability of MASTL to enhance metastasis has to our knowledge, not been previously reported, and likely explains the poor DMFS seen in breast cancer patients that overexpress MASTL. The ability of MASTL knockdown to delay both primary tumour growth and prevent metastasis suggests that inhibiting MASTL may be a valid therapeutic strategy for the treatment of breast cancer, which is also supported by another manuscript that was published during the revision of our paper [16]. Excitingly, a first-generation small molecule inhibitor for MASTL was recently identified [45], and the utility of second-generation inhibitors as targeted chemotherapeutics will be of great interest. In summary, we confirm previous reports that MASTL overexpression disrupts PI3K/ATK/mTOR signalling, which over-comes contact inhibition, drives a partial EMT phenotype allowing uncontrolled proliferation. Furthermore, we identified a novel mechanism whereby MASTL overexpression disrupts the timing of mitotic exit resulting in increased chromosome segregation defects and micronuclei formation. Taken together, these results support the role that MASTL is a novel breast cancer oncogene capable of driving CIN, invasion and metastasis, which results in more aggressive breast tumours and reduced patient survival.

## Materials and methods

### Chemicals, reagents and antibodies

The following chemicals were used: “heavy” lysine-^13^C_6_^14^N_2_ (lys8) (Silantes (GmBH), arginine-^13^C_6_^14^N_4_ (arg10) (Silante, GmBH), 1.9 µm C18 AQ particles (Dr. Maisch), 1,1,1-Trifluoro-Ethanol (Sigma), Iodoacetimide (Sigma), TCEP (ThermoFisher) Tetrafluoro Acetic Acid (Optima-grade, Fluka), Acetonitrile (ACN) (Optima-grade, Fluka), ammonium hydroxide (HPLC-grade, Sigma), SB203580 (SelectChem), Doxycyline, H33342 and DAPI (Sigma). All antibodies used are described in Supplemental Table S2.

### MASTL overexpression and shRNA

Stable clones were generated by lentiviral infection using pReceiver-Lv213 vectors without any insert (Empty vector, EV control) or containing the full protein-coding open reading frame of human MASTL transcript variant 1 (NM_001172303.1). EV and MASTL vectors were purchased from Genecopoeia (catalogue #EX-NEG-Lv213 and #EX-T8386-Lv213 respectively). Stable clones were generated by lentiviral infection using SMARTvector Inducible Human MASTL mCMV-TurboGFP shRNA purchased from Dharmacon (#V3SH7669-225461809), with a mature antisense sequence of 5ʹ-CTGATAAGCGATAACACTT-3ʹ, corresponding to 629–647 of the full CDS for MASTL (NM_001172303).

### IHC and mice methods

Immune-compromised NOD Scid IL2 gamma–/– mice were housed in specific-pathogen-free conditions in a 12-h:12-h light:dark cycle and given food and water ad libitum. DOX-containing food (700 mg/kg) was purchased from Gordon’s Specialty Stock Feeds and replaced weekly. For orthotopic assays, 5 × 10^6^ MDA-MB-231 cells stably expressing H2B-mCherry and either shControl of shMASTL-inducible vectors were injected subcutaneously into mammary fat pads. Mice were randomised onto DOX or control diets. Mice were weighed and tumours measured twice weekly. When tumours reached ethical endpoint (10% tumour burden), mice were euthanised with CO_2_ asphyxiation, in accordance with all ethical regulations (approval #ARA 17_23). Fresh tissue was snap frozen, then lysed with stainless steel beads on TissueLyser (Qiagen) in RIPA buffer. For Intraductal (MIND) Xenograft assays, intraductal injections were carried out as described previously [46]. Briefly, 80,000 MDA-MB-231 cells stably expressing H2B-mCherry and either shControl of shMASTL inducible vectors were injected in 4 µl into mammary ducts via nipple incision. All mice were fed a DOX diet and monitored twice weekly for weight and tumour size. Mice were euthanised at 7 weeks by CO_2_ asphyxiation. Mammary glands were fixed in 10% neutral buffered formalin (NBF) overnight and stained with carmine. Following imaging of whole mounts, mammary glands were paraffin embedded. Lungs were inflated and fixed for 12 h in 10% NBF. All tissues were then paraffin embedded, then stained with hematoxylin and eosin for routine histochemistry or stained with anti-mCherry using Envision immunohistochemistry on the Leica BOND as per the manufacturer’s instructions. Images were taken with Aperio ScanScope Slide Scanner.

## Electronic supplementary material

Supplemental Material and Methods

Supplemental Text and Figures

Table S1

Table S2

Figure S1

Figure S2

Figure S3

Figure S4

Figure S5

Figure S6

Figure S7
