# Supplementary material for: MASTL overexpression promotes chromosome instability and metastasis in breast cancer
Source: Oncogene. 2018 May 10;37(33):4518–33. doi: 10.1038/s41388-018-0295-z (PMC6095835; doi:10.1038/s41388-018-0295-z)
Supplement: Supplementary file 1 — Supplemental Material and Methods [file 41388_2018_295_MOESM1_ESM.docx]

**Supplemental Experimental Procedures**

***Immunofluorescence and live cell imaging***

Cells were grown on Histogrip (Life Technologies) coated glass coverslips and fixed with 3.7% formaldehyde diluted in PHEM buffer (60 mM Pipes, 25 mM hepes, 1 mM EGTA, 2 mM MgCl2) with 0.5% Triton X-100 for 10 min. All cells were washed and then blocked (3% BSA, 0,1% Tween 20 in PBS) for 30 min. Cells were incubated with primary antibodies were incubated for 2 h at room temperature in blocking solution. DNA was stained with H33342 and imaged using a Leica DMI5500 (40x, 100x) or DM6000 SP8 confocal with a 63× lens. In some cases, 0.3 µm Z-sections were taken and displayed as 2D slices or maximum projections using Fiji (Image J v1.51n) and compiled using Adobe Photoshop CC 2017 software. 3D renderings and 3D volume, and ellipticity analysis were performed using Imaris (v8) software. Live cell imaging and IncuCyte proliferation assays were performed as previously described ^1^. Briefly, for live cell imaging, cells were seeded at 35% confluence on 6 or 12 well plates and imaged using a Leica DMI6000 using a 20X NA 0.4 objective. Images were taken every 2-3 min for 48 h. Individual cells were followed and scored for nuclear envelope breakdown (NEBD) and first signs of anaphase. Mitotic length = NEBD to anaphase, while interphase length = anaphase to next daughter cell NEBD. Only the first daughter cell to divide was followed and annotated. For wound healing assays, cells were seed at confluence in Ibidi 2-well culture-inserts place in 12-well plates. 24 h later inserts were removed and cells were imaged as per live imaging, at 10min intervals. MRI Wound Healing Tool, MTrackJ ^2^ and DiPer ^3^ were used to determine wound area, individual cell speed and directionality ratios. For IncuCyte assays, cells were seeded on 24 well plates and filmed for up to 4 days at 4 h intervals. Confluence and nuclear masks (for MDA-MB-231 H2B-mCherry cells) were generated and used to determine cell proliferation as previously described ^1^

***Phospho-AKT MagPix Assay***

MagPix assays were performed using the manufactures protocol. Briefly. EV-control and MASTL cells were pelleted and frozen at -80 ºC, cells were lysed using Cell Signalling Lysis Buffer II (Merck Millipore), clarified at 13,000 rpm for 20 min at 4 ºC and equalised for protein by BCA assay. 250 µg of protein was bound overnight at 4 ºC to the bead solution provided with the 11-plex milliplex MAP phospho AKT/mTOR magnetic bead kit (Merck Millipore). Washing and phycoerythrin amplification steps were performed using the Bio-Plex Pro Wash Station (Biorad). Phycoeyrthrin signal was detected using the Luminex MAGPIX instrument with xPONENT software (v4.2). All samples were performed as 3 independent biological replicates, and 2 technical replicates to account for instrument variation. Assays recorded on different days were normalised using the average background intensity.

***Western blot and RT-PCR***

Proteins from whole-cell lysates were resolved under reducing conditions on 4–12% Bis-Tris polyacrylamide gels (Life Technologies) using standard methods. Resolved proteins were transferred to 0.2 μm PVDF membranes (BioRad) and incubated with the indicated antibodies overnight at 4 °C. Protein bands were detected by appropriate HRP-conjugated secondary antibody and detected using western lightning ECL reagent (Perkin-Elmer) or ClarityMAX (BioRad) on a ChemiDoc Touch (BioRad). Blots were checked for equal loading by re-probing with anti-β-actin, or anti-GAPDH. Densitometry was measured using GelEval software (v 1.37). For RT-PCR, total RNA was isolated using an RNeasy kit (Qiagen) and was reverse-transcribed using the Reverse Transcription System (Promega) according to the manufacturer’s instructions. Real-time PCR was performed using an ABI Prism 7900HT sequence detection system (Life Technologies) using inventoried Taq-Man probes (Life Technologies) for MASTL (Hs00949171_m1) and RPLPO (Hs99999902_m1) (loading control). Data analyses were performed using the ΔΔCt method, as previously described ^4^. Fold changes in gene expression were calculated relative to normal breast cell lines.

***3D Cell Culture and Organotypic invasion assays***

MatriGel (BD Corning) was defrosted overnight at 4 ^o^C, 8 well chamber slides (Falcon) were coated with matrigel, and then allowed to solidify at 37 ^o^C for 30 min. Cells were seeded in their respective media (for MCF10A, EGF was reduced to 5 ng/mL, and serum to 2%) containing 2% matrigel, replacing media every 4 days. Once colonies reached endpoint matrigel was fixed in 4% PFA in PBS containing 1% glutaraldehyde for 10 min at RT, quenching the glutaraldehyde with 100 mM glycine. Following fixation, all chamber slides were processed and imaged within 48 h, as per immunofluorescence staining.

3D organotypic invasion assays were performed as previously described ^5^. Briefly, ~ 3 x 10^5^/ml primary fibroblasts were embedded in a three-dimensional matrix of rat tail collagen І. Rat tail tendon collagen solution was prepared by the extraction of tendons with 0.5 M acetic acid to a concentration of ~ 2 mg/ml. Detached, polymerized matrix (2.5 ml) in 35 mm petri dishes was allowed to contract for approximately 12 days in complete media (DMEM, supplemented with 10% foetal bovine serum, Invitrogen) until the fibroblasts had contracted the matrix to ~1.5 cm diameter. Subsequently, 1 x 10^5^ MDA-MB-231 cells were plated on top of the matrix in complete media (+/- 2 µg/mL doxycycline) and allowed to grow to confluence for 4 days. The matrix was then mounted on a metal grid and raised to the air/liquid interface resulting in the matrix being fed from below with complete media including doxycycline was changed every 3 days. After 14 days, the cultures were fixed using 10% formalin and processed by standard methods for immunohistochemistry with hematoxylin and eosin (H&E), and multi-cytokeratin staining. Individual cells that had invaded into the collagen were counted, values reflect the percentage of cell invaded, compared with the number of cells that remained in a monolayer closest to the surface of the collagen matrix.

***DNA Fibre Analysis***

Briefly, normally growing MCF10A cells were pulse labelled in growth media with 50 µM IdU (in replacement of thymidine) for 30 min washed 2x with pre-warmed PBS, and then replaced in media containing 50 µM CldU for 30 mins. Following 2x washes in ice-cold PBS, cells were harvested with trypsin, centrifuged, and resuspended at a concentration of 0.5 x 106 cell / 45 µL. Equi-volume amounts of melted 1.2 % LMT agarose was mixed with the cell suspension and pipetted into agar reservoirs placed at 4 C to set. Cellular proteins contained in the solidified agar plugs were digested in ESP buffer (0.5 M EDTA pH 8.0, 1% Sarcosyl/EDTA, 4 mg/mL Proteinase K) overnight at 50 C. The following day, plugs were washed 3x with TE buffer, before the agar was digested overnight in 0.5 M MES pH 5.5 containing 1 U agarase overnight at 42 C. The resultant DNA solution was then combed onto CombiCoverslips (Genomic Vision) using a molecular combing apparatus (Genomic Vision). Following 2 h incubation at 68 C, slides were dehydrated in baths progressing from 70-100 % EtOH. The remaining steps were carried out as per the immunofluorescence method, IdU was probed with rat anti-BrdU antibodies (BD), and CldU was probed with anti-BrdU antibodies (AbCam). Automated computer aided scoring and analysis (CASA) DNA Fibre analysis performed by Paul Chastain as previously published ^6^.

***SILAC Labelling and Phospho-Enrichment***

Briefly, 10A cell lines were cultured in SILAC F12:DMEM (1:1) (Sigma) deficient in lysine and arginine. MCF-10A cells were cultured as previously described ^7^, for at least 6 doublings in media containing “heavy” lysine-^13^C_6_^14^N_2_ (Lys8), and arginine-^13^C_6_^14^N_4_ (Arg10) or the respective “light” counterpart (Lys0, Arg0), amino acid incorporation was checked routinely. Protein was harvested by scraping in GdmCl buffer (6 M GdmCl, 100 mM Tris pH 8.5, 10 mM TCEP, 60 mM IAM). Heavy and light samples were then mixed 1:1. Whole cell extracts were diluted 50% in milli-Q H_2_O and precipitated overnight at -20 in 4x Volumes of acetone. Precipitated proteins were digested in Lys-C, and trypsin at 100:1 (w:w) overnight at 37 ^o^C. The resultant mixture was then enriched for phosphorylated peptides using the EasyPhos method as described ^8^. Briefly, phospho-peptides were bound to TiO_2_ beads (5 µm Titanosphere, GL Bioscience) at a ratio of 10:1 (protein:beads) at 40 ºC for 5 mins, after washing (80% ACN, 0.5% acetic acid), phosphopeptides were eluted (40% ACN, 15% Ammonium Hydroxide), dried, and loaded onto SDB-RPS StageTips (3M empore), and then eluted (80% ACN, 5% ammonium hydroxide), dried, and stored at -80 ºC until mass spectrometry analysis.

***Mass Spectrometry, Data Analysis, and Bioinformatics***

Digested phospho-peptides were analysed as previously described ^9^. Briefly, a 50 cm x 75 μm fused silica column, packed in-house using 1.9 μM C18AQ particles using an Easy nLC-1200. Peptides were separated using a 195 min gradient using a binary buffer system of Buffer A (0.1% formic acid) and Buffer B (80% ACN, 0.1% formic acid) at a flow rate of 300 nL/min. Peptides were eluted with a gradient of 5-30% buffer B over 150 min, followed by 30-60% buffer B over 5 min, and 60-95% buffer B over 5 mins. Peptides were analysed on a Q-Exactive HF mass spectrometer operated in positive-ion DDA mode, with one full scan (300-1,650 m/z, *R*=35,000 at 200 m/z), at a target of 3e^6^, selecting top 20 most abundant precursor ions (isolation window = 1.4 m/z, ion target 3e^5^) for HCD fragmentation (NCE=27%) and MS2 scan (*R*=35,000 at 200 m/z) ^9^. Thermo RAW files were generated in centroid mode and analysed using MaxQuant (v.1.5.3.3) with integrated Andromeda search engine, searching against a whole proteome, with additions (uniprot release 01.2016) ^10^. Default MaxQuant settings were used, with 1% FDR and the addition of match between runs and matching with an elution time of 1.0 min. Bioinformatics were performed in Perseus (1.5), and Microsoft Excel (2010) ^11^. Briefly, SILAC H/L ratios determined by MaxQuant were inverted for label-swaps, Log_2_ transformed prior to statistical analysis. Non-significant Log_2_H/L ratios were eliminated using the Student’s t-test, with P<0.05 as a significance cut off, ratios >1, or <-1 considered significantly altered. KEGG pathway enrichments were determined from the parent protein identified from the individual phospho-peptides using the Enrichr Online Tool ^12^. Interaction networks were produced using StringDB and the ClueGO plugin of Cytoscape (v3.3).

***Cell Culture, Patient Cohort and Public Database Analysis***

MCF10A cells were purchased from Sigma, MDA-MB-231 cells were purchased from the ATCC. Production of H2B-mCherry stable cell lines was performed as previously described ^1^. MCF10A and MDA-MB-231 cells were cultured at 37 ^o^C, 5% CO_2_. MCF10A were cultured in DMEM/F12 (1:1) supplemented with 5% horse serum, 20 ng/mL of recombinant human EGF, 0.5 μg/mL hydrocortisone, 100 ng/mL cholera toxin, 10 μg/mL bovine insulin as described previously ^7^. MDA-MB-231s were cultured in RPMI supplemented with 10% foetal bovine serum, 0.25 U/mL human insulin, and L-Glutamine. All cells were validated to >90% match at CellBank, or ATCC by STR profiling, and routinely checked for mycoplasma contamination. Breast cancer patient tumours Cases were drawn from the St. Vincent’s Campus Outcome Cohort and previously described ^13^. CPTAC analysis was performed in Matlab as described ^14^. TCGA dataset analysis was performed using cBioPortal (default setting) ^15^, and survival analysis was performed using KmPlot ^16^.

***Mathematical modelling and Statistics***

The mathematical model of mitotic exit, was generated and used as previously described ^7^. Briefly, we developed a computational model based on our empirical data to delineate the minimal requirements needed for triggering PP1 dephosphorylation of MASTL and mitotic exit. A central component for mitotic exit is the presence of a bistable switch (green shading), where the presence of feedback loop/s trigger the transition between two stable states, mitosis (on, red shading) and interphase (off, blue shading) (Fig. 5A). The core of our model is based on established models of Cdk1, MASTL, ENSA and PP2A. Specifically, our model comprises two feed-forward loops (black lines) from CDK1, the first suppresses the phosphatase activities of PP1 by phosphorylation of Thr320 and the second suppresses PP2A via MASTL phosphorylation of ENSA. Two feedback loops (red lines) were also incorporated; a positive feedback loop from PP1 to itself and a double negative feedback loop via inhibition of MASTL and re-activation of PP2A as indicated by our data. In the positive loop, PP1 can activate itself through auto-dephosphorylation of its inhibitory Thr320, which is under the control of Cdk1. The double negative feedback loop is initiated by the deactivation of MASTL by PP1, which in turn releases PP2A inhibition. All other mathematic operations, and statistics were performed using Microsoft Excel (2016), and Prism (v 7.0c)

**Supplemental References**

1 McCloy RA, Rogers S, Caldon CE, Lorca T, Castro A, Burgess A. Partial inhibition of Cdk1 in G 2 phase overrides the SAC and decouples mitotic events. *Cell Cycle* 2014; **13**: 1400–1412.

2 Bettencourt-Dias M, Giet R, Sinka R, Mazumdar A, Lock WG, Balloux F *et al.* Genome-wide survey of protein kinases required for cell cycle progression. *Nature* 2004; **432**: 980–987.

3 Gorelik R, Gautreau A. Quantitative and unbiased analysis of directional persistence in cell migration. *Nat Protoc* 2014; **9**: 1931–1943.

4 Roberts CG, Millar EKA, O'Toole SA, McNeil CM, Lehrbach GM, Pinese M *et al.* Identification of PUMA as an estrogen target gene that mediates the apoptotic response to tamoxifen in human breast cancer cells and predicts patient outcome and tamoxifen responsiveness in breast cancer. *Oncogene* 2011; **30**: 3186–3197.

5 Vennin C, Chin VT, Warren SC, Lucas MC, Herrmann D, Magenau A *et al.* Transient tissue priming via ROCK inhibition uncouples pancreatic cancer progression, sensitivity to chemotherapy, and metastasis. *Sci Transl Med* 2017; **9**. doi:10.1126/scitranslmed.aai8504.

6 Wang Y, Chastain P, Yap P-T, Cheng J-Z, Kaufman D, Guo L *et al.* Automated DNA fiber tracking and measurement. IEEE, 2011, pp 1349–1352.

7 Rogers S, Fey D, McCloy RA, Parker BL, Mitchell NJ, Payne RJ *et al.* PP1 initiates the dephosphorylation of MASTL, triggering mitotic exit and bistability in human cells. *J Cell Sci* 2016; **129**: 1340–1354.

8 Humphrey SJ, Azimifar SB, Mann M. High-throughput phosphoproteomics reveals in vivo insulin signaling dynamics. *Nat Biotechnol* 2015; **33**: 990–995.

9 Hoffman NJ, Parker BL, Chaudhuri R, Fisher-Wellman KH, Kleinert M, Humphrey SJ *et al.* Global Phosphoproteomic Analysis of Human Skeletal Muscle Reveals a Network of Exercise-Regulated Kinases and AMPK Substrates. *Cell Metabolism* 2015; **22**: 922–935.

10 Cox J, Neuhauser N, Michalski A, Scheltema RA, Olsen JV, Mann M. Andromeda: a peptide search engine integrated into the MaxQuant environment. *J Proteome Res* 2011; **10**: 1794–1805.

11 McCloy RA, Parker BL, Rogers S, Chaudhuri R, Gayevskiy V, Hoffman NJ *et al.* Global Phosphoproteomic Mapping of Early Mitotic Exit in Human Cells Identifies Novel Substrate Dephosphorylation Motifs. *Mol Cell Proteomics* 2015; **14**: 2194–2212.

12 Chen EY, Tan CM, Kou Y, Duan Q, Wang Z, Meirelles GV *et al.* Enrichr: interactive and collaborative HTML5 gene list enrichment analysis tool. *BMC Bioinformatics* 2013; **14**: 128.

13 López-Knowles E, O'Toole SA, McNeil CM, Millar EKA, Qiu MR, Crea P *et al.* PI3K pathway activation in breast cancer is associated with the basal-like phenotype and cancer-specific mortality. *Int J Cancer* 2010; **126**: 1121–1131.

14 Fey D, Halasz M, Dreidax D, Kennedy SP, Hastings JF, Rauch N *et al.* Signaling pathway models as biomarkers: Patient-specific simulations of JNK activity predict the survival of neuroblastoma patients. *Sci Signal* 2015; **8**: ra130–ra130.

15 Gao J, Aksoy BA, Dogrusoz U, Dresdner G, Gross B, Sumer SO *et al.* Integrative analysis of complex cancer genomics and clinical profiles using the cBioPortal. *Sci Signal* 2013; **6**: pl1.

16 Győrffy B, Surowiak P, Budczies J, Lánczky A. Online survival analysis software to assess the prognostic value of biomarkers using transcriptomic data in non-small-cell lung cancer. *PLoS ONE* 2013; **8**: e82241.
