# Supplementary material for: MASTL overexpression promotes chromosome instability and metastasis in breast cancer
Source: Oncogene. 2018 May 10;37(33):4518–33. doi: 10.1038/s41388-018-0295-z (PMC6095835; doi:10.1038/s41388-018-0295-z)
Supplement: Supplementary file 2 — Supplemental Text and Figures [file 41388_2018_295_MOESM2_ESM.docx]

**Supplemental Text and Figures**

**Supplemental Figure S1**. High MASTL expression correlates with poorer overall survival in cancer. **(A)** Cross-cancer MASTL mutations currently annotated in TCGA provisional dataset. **(B-C)** The correlation between high MASTL expression and poorer survival from various parameters using the Kaplan Meier-plotter database (KmPlot) for **(B)** Breast cancer **(C)** Lung and Ovarian cancer. **(D)** Kaplan Meier curves for for MASTL protein expression from the CPTAC database **(E)** The spectrum of alterations (amplification, deletion, upregulation, downregulation, and mutation) of MASTL compared with the breast cancer susceptibility gene (BRCA1) in breast cancer tumours within the 2016 METABRIC TCGA dataset (cBioPortal). Alterations of both genes are seen in 9% of all breast cancer tumours within the cohort. The amplification, and upregulation of MASTL is most prominent in the basal / triple negative breast cancer (TNBC) subtype, where 44% of tumours are altered (inset). **(F)** Western blot analysis of MASTL expression across normal (blue shading) and breast cancer (red shading) cell lines, including actin (loading control).

**Supplemental Figure S2**. MASTL over-expression increases the presence of mitotic defects. **(A)** Western blot analysis of asynchronously growing MCF10A cells stably expressing either an empty vector (Cont) or full-length MASTL. **(B)** Representative phase contrast images of a normal mitosis, compared to defective mitosis where out of plane cleavage, cleavage defects, or multipolar division has occurred. These defects were quantified in both cell lines by live-cell imaging and reflected as % of total mitoses (Unpaired students t-test, * P<0.05, **P< 0.001). **(C)** Representative microscopy field used for threshold image analysis on the H33342 channel. Nuclei were masked for analysis (blue nuclei), while those on the edge of the field were excluded from the particle counting (white nuclei). This analysis indicated an increased relative frequency of nuclei >225 µm^2^, and significantly more micronuclei per field in the MASTL over-expressing cells (mean±SEM, unpaired students t-test, *P<0.05). **(D)** MASTL over-expressing, and EV-control cells were fixed and stained for H33342 (cyan), TPX2 (red), phalloidin (yellow). The presence of anaphase chromatin bridges (inset) were quantified and expressed as a % of total mitoses counted (n= ???, unpaired students t-test, * P<0.05)

**Supplemental Figure S3**. MASTL over-expression increases the phosphorylation of proteins involved in hemidesmosome signalling. **(A)** 5 replicates of SILAC labelled EV-control and MASTL cell lines were analysed by mass spectrometry, values indicate the correlation in the H/L ratio of the peptides identified in all replicates. **(B)** Venn diagram indicating there is no overlap in the proteins present in either the UP or DOWN groups **(C)** ClueGO enrichment analysis of the UP (red) and DOWN (blue) proteins into ontology terms. Relative size of the node is indicative of the enrichment within the dataset, edge weight indicates the level of confidence in the interaction between ontology terms. **(D)** Group Based Prediction (GPS3.0) of the upstream kinases responsible for phosphorylating the proteins in the UP (red bars) and DOWN (blue bars) group, expressed as a % of the total number of phosphorylations. Kinases that had significantly changed enrichment between the UP and DOWN group are highlighted.

**Supplemental Figure S4**. MASTL expression correlates with PI3K/AKT/mTOR pathway deregulation in breast cancer cell lines and patient samples. (A) MASTL comparative Protein expression array (RPPA) analysis of the Provisional TCGA Breast Invasive Carcinoma dataset (892 samples). **(B)** Cytoscape ClueGO enrichment analysis of significantly co-over and co-under expressed proteins from A. (inset) Mutational enrichment analysis for dataset in A. **(C)** Correlation of MASTL protein expression with various proteins by TMA in our cohort of Breast tumour samples. **(D)** Western blot analysis of a panel of breast cancer cell lines. Protein was normalised to GAPDH loading, expressed relative to MCF10A and correlated to MASTL protein levels.

**Supplemental Figure S5**. MASTL over-expression removes contact inhibition, and results in aberrant β-catenin localisation. **(A)** Focus formation assay of the MASTL and EV-control cell lines, crystal violet staining indicates that MASTL over-expressing cells that have grown in densely overlapping foci. **(B)** MASTL over-expressing cells were fixed and stained with H33342 (cyan), phalloidin (magenta), and β-catenin (yellow), to show the aberrant localisation of β-catenin. **(C)** Western blot analysis for β-catenin (β-cat), E-Cadherin (E-Cad), MASTL and GAPDH (loading), of asynchronous MCF10A control or MASTL over-wexpressing cells**. (D)** Additional images of 3D cultured MCF10A cells presented in Figure 5D. 7-day acini were fixed and stained with H33342 (cyan) and phalloidin (red) and analysed by confocal microscopy with 0.3 µm z-stacks from the top of the acini to bottom, the acini were reconstructed in 3D, and rendered in imaris (3D-render).

**Supplemental Figure S6**. Inhibition of p38 rescues MASTL induced cell cycle delays. (A) Single-cell fate maps for MCF10A cells expressing empty vector (Control) or MASTL. Asynchronous cells were followed by time-lapse microscopy, with images taken every 2-3 min for 48 h. (B) Histogram charting the number of cell divisions (percentage) in 48 h. (C) Migration analysis of cells in A, by MTrackJ. The DiPer analysis program was used to generate centred plots for all tracks.

**Supplemental Figure S7**. Knockdown of MASTL causing mitotic defects and reduced cell proliferation in MDA-MB-231 breast cancer cells. **(A)** No significant difference was observed between the length of interphase between mother and daughter cells (1-way ANOVA). **(B)** Single-cell fate maps for MDA-MB-231 cells treated with or without Dox to induce shRNA expression. **(C)** Quantification of mitotic defects observed in B. **(D)** Still images from B. showing examples of the mitotic defects observed. **(E)** Histogram charting the number of cell divisions (percentage) completed in 48 h.
