# Supplementary material for: MASTL overexpression promotes chromosome instability and metastasis in breast cancer
Source: Oncogene. 2018 May 10;37(33):4518–33. doi: 10.1038/s41388-018-0295-z (PMC6095835; doi:10.1038/s41388-018-0295-z)
Supplement: Supplementary file 4 — Table S2 [file 41388_2018_295_MOESM4_ESM.pdf]

Supplementary Table S2. All primary, secondary, and cojugated antibodies used in this manuscript.

Antibody	Species	Supplier	Catalogue #	Dilution Factor
MASTL (Full Length)	Rabbit	Kind Gift A.Castro	n/a	500(IF) 1000(WB)
HRP-linked Donkey anti-Rabbit IgG Antibody	Rabbit	GE Life Sciences	NA934	1000
HRP-linked Sheep anti-Mouse IgG Antibody	Mouse	GE Life Sciences	NA931	1000
β-Actin	Goat	Santa Cruz	sc-1616	400
ß-Tubulin	Mouse	Gift from Natalie Morin	n/a	1:50 (IF)
Alexa Fluor 488 Goat anti-Rabbit IgG Antibody	Goat	Life Technologies	R37116	80
Alexa Fluor 594 Donkey anti-Mouse IgG Antibody	Donkey	Life Technologies	R37115	80
pChk1 (S345) (133D3)	Rabbit	Cell Signalling Technology	2348	1000
Pan-Cytokeratin	Mouse	Leica-Novocastra	NCL-C11	50
GAPDH	Mouse	Santa Cruz	SC-32233	2000
mTOR (7C10)	Rabbit	Cell Signalling Technology	2983	1000
phospho-mTOR (Ser2481)	Rabbit	Cell Signalling Technology	2974	1000
Phospho-P38α (Thr180/Tyr182)	Rabbit	Cell Signalling Technology	4511	1000
P38α (D13E1)	Rabbit	Cell Signalling Technology	8690	1000
Phospho-Histone H2A.X (Ser139) (20E3)	Rabbit	Cell Signalling Technology	9718	1000
RPS6 (54D2)	Mouse	Cell Signalling Technology	2317S	1000
phospho-RPS6 (Ser240/244) (D68F8) XP	Rabbit	Cell Signalling Technology	2215	1000
Phalloidin iFluor-647 CytoPainter	n/a	ABCAM	ab176759	1000
RFP	Rabbit	Life Research (Rockland)	600-401-379	1000
E-Cadherin	Rabbit	Epitomics/AbCam	10702-1/ab40772	500(IF) 1000 (WB)
β-Catenin	Mouse	BD Biosciences	BD610153	250(IF) 1000 (WB)
BrdU (BrdU & IdU)	Mouse	BD Biosciences	347580	5
BrdU [BU1/75 9ICR1)] (BrdU & CldU)	Rat	ABCAM	ab6326	25
Alexa Fluor 488 Goat anti-Mouse IgG Antibody	Goat	Life Technologies	A-11001	25
Alexa Fluor 594 Goat anti-Rat IgG Antibody	Goat	Life Technologies	A-11007	25
TPX2 (D2R5C) XP	Rabbit	Cell Signalling	12245	1000

(WB) = Western Blot (IF) = immunofluorescence
