# Supplementary figures and images for: MASTL overexpression promotes chromosome instability and metastasis in breast cancer
Source: Oncogene. 2018 May 10;37(33):4518–33. doi: 10.1038/s41388-018-0295-z (PMC6095835; doi:10.1038/s41388-018-0295-z)

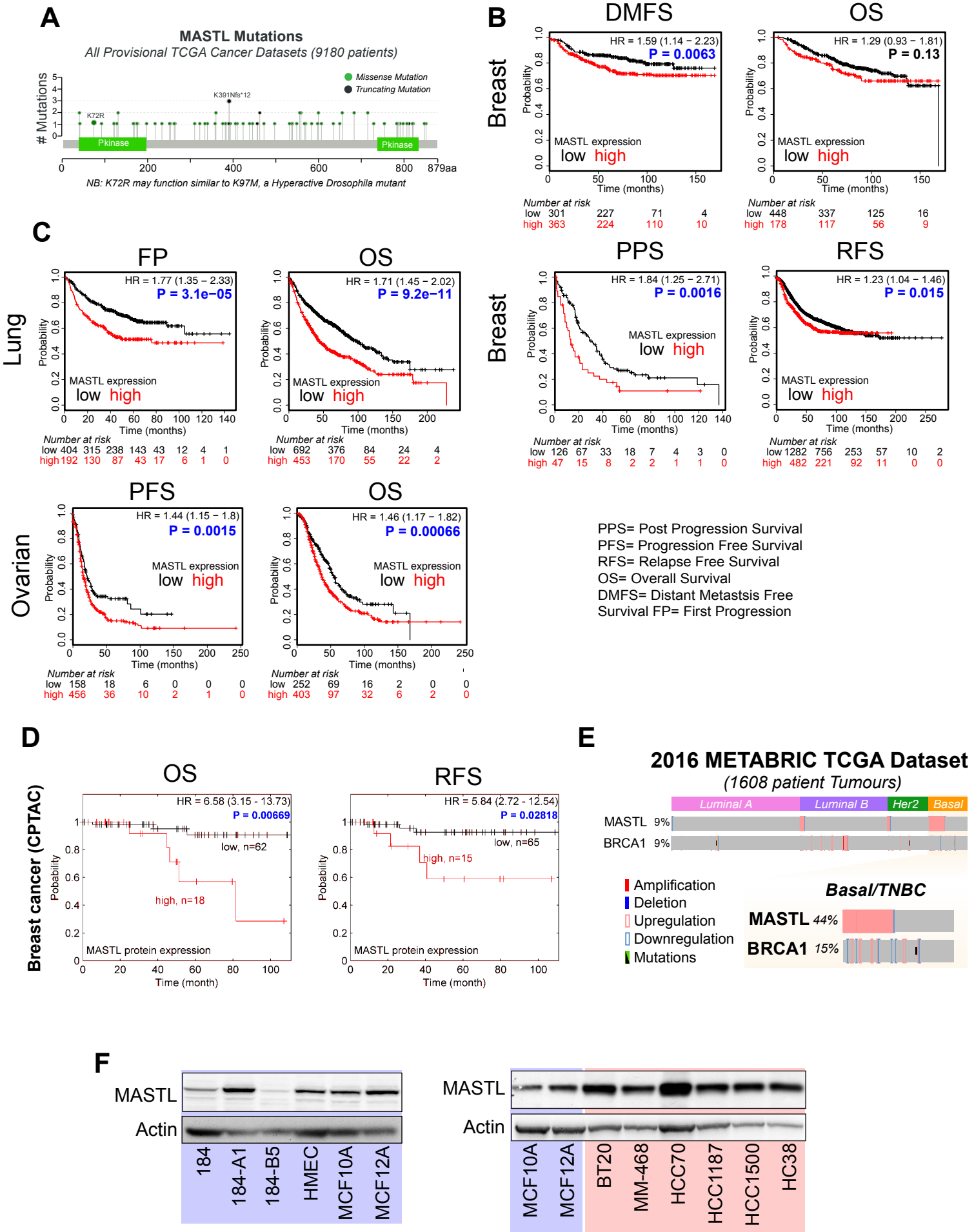

Supplement: Supplementary file 5 — Figure S1 [file 41388_2018_295_MOESM5_ESM.pdf]

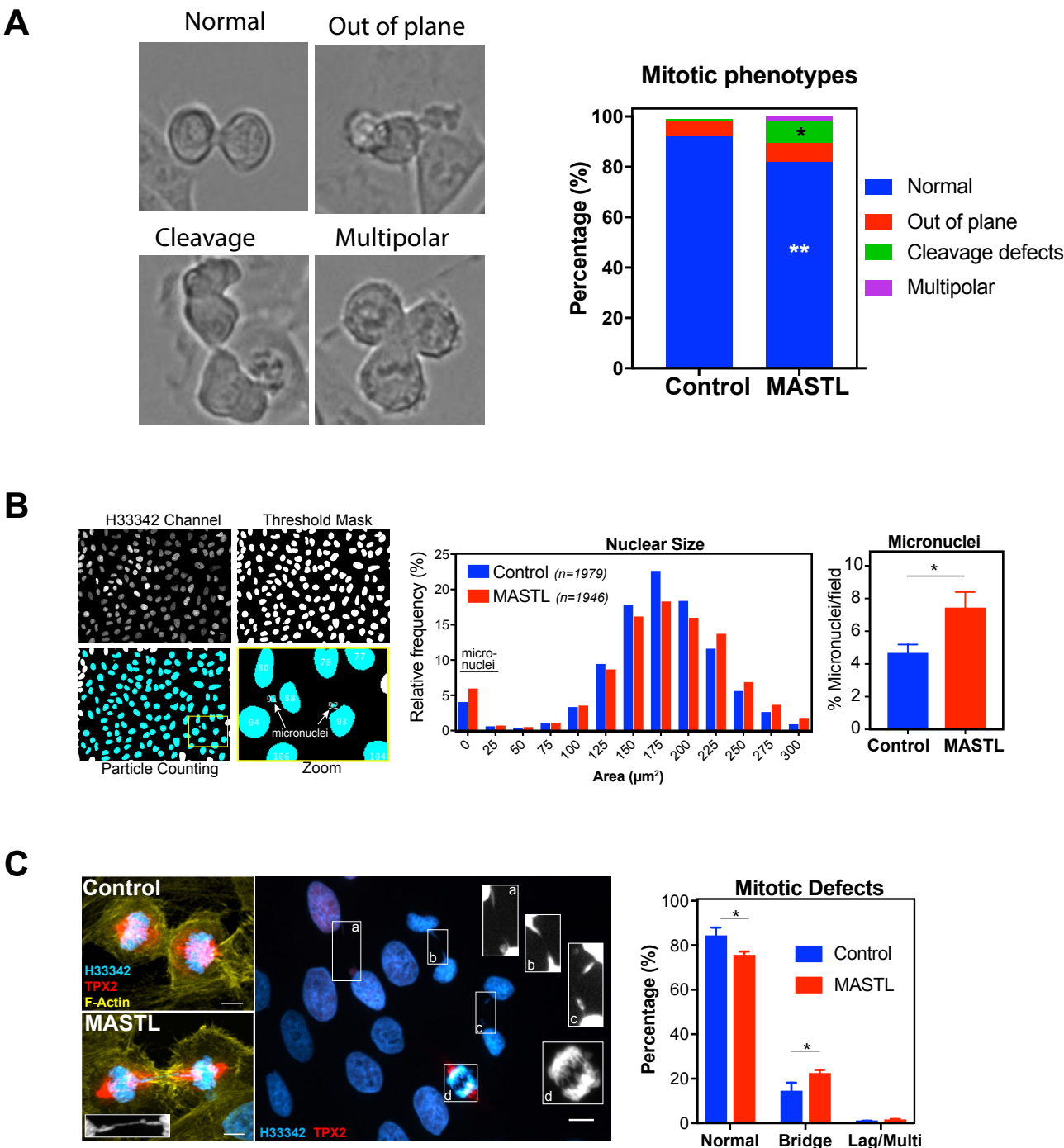

Supplement: Supplementary file 6 — Figure S2 [file 41388_2018_295_MOESM6_ESM.pdf]

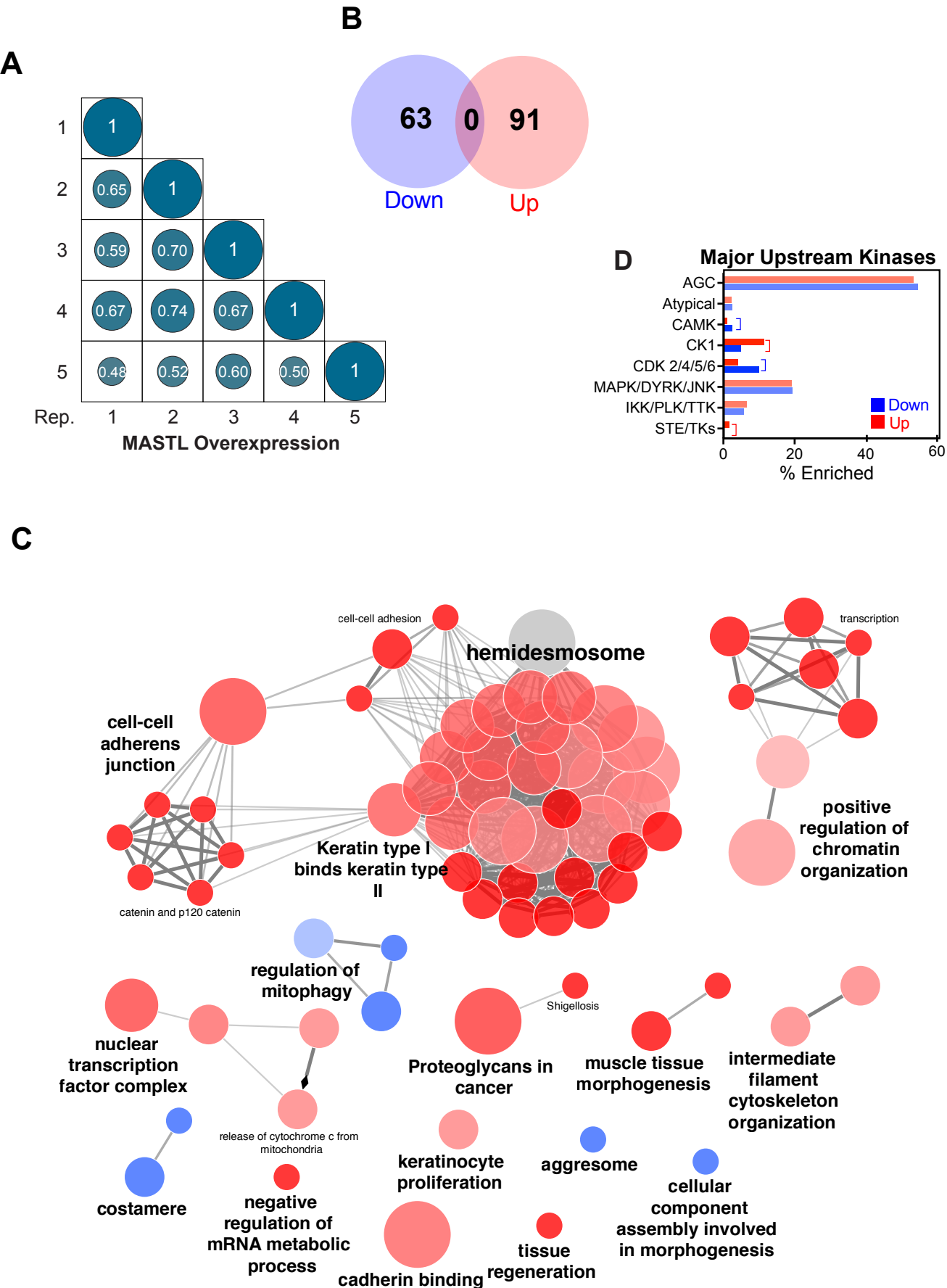

Supplement: Supplementary file 7 — Figure S3 [file 41388_2018_295_MOESM7_ESM.pdf]

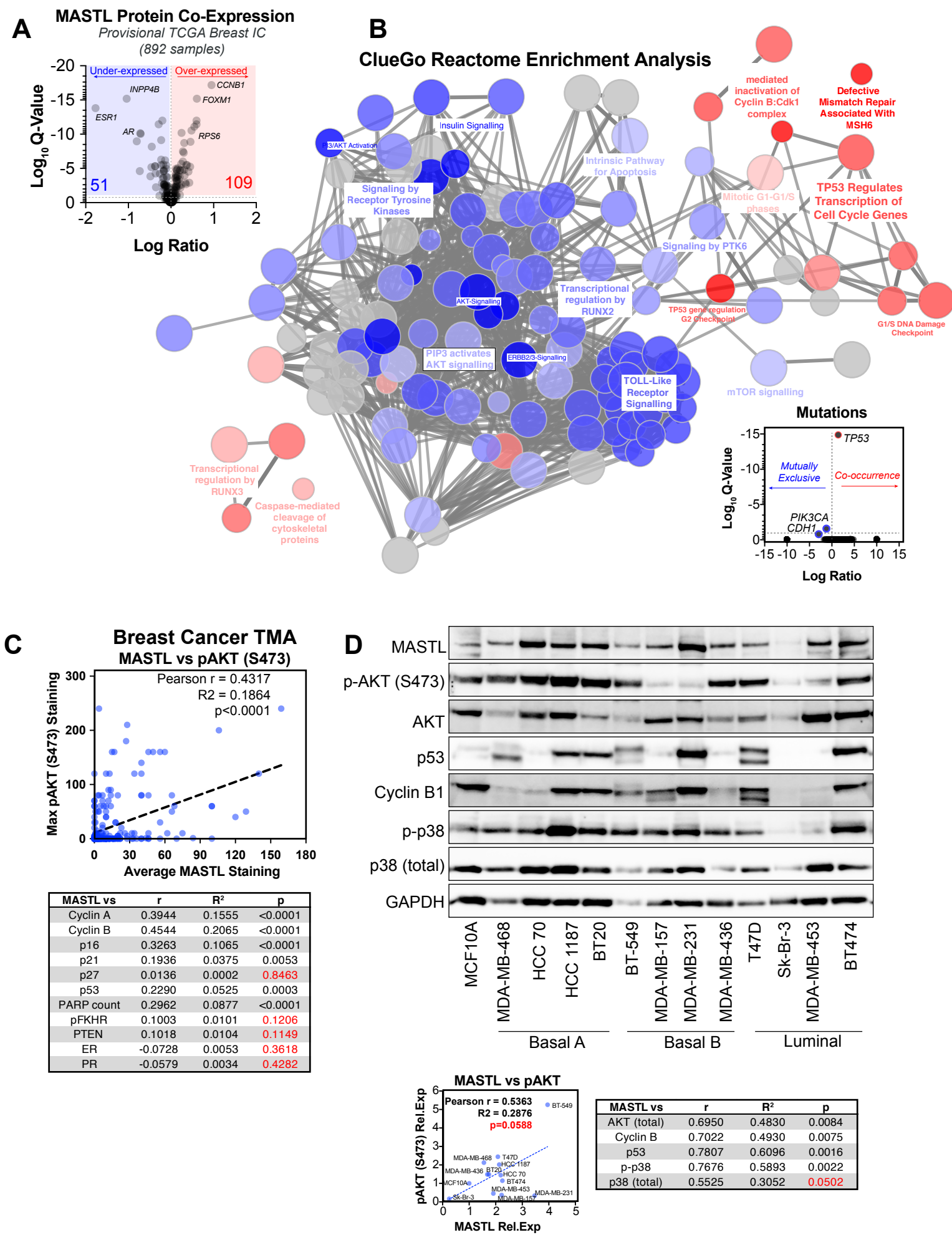

Supplement: Supplementary file 8 — Figure S4 [file 41388_2018_295_MOESM8_ESM.pdf]

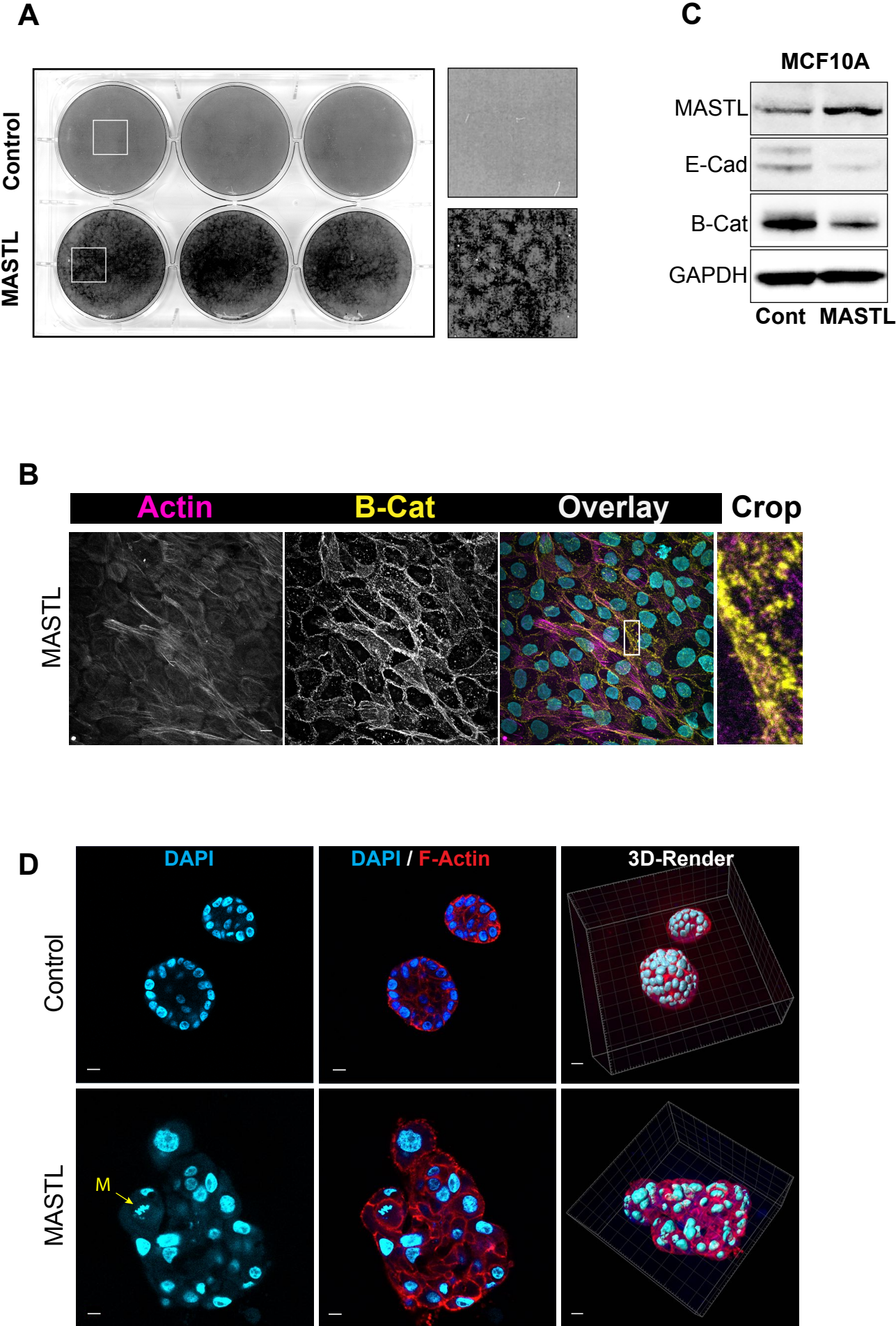

Supplement: Supplementary file 9 — Figure S5 [file 41388_2018_295_MOESM9_ESM.pdf]

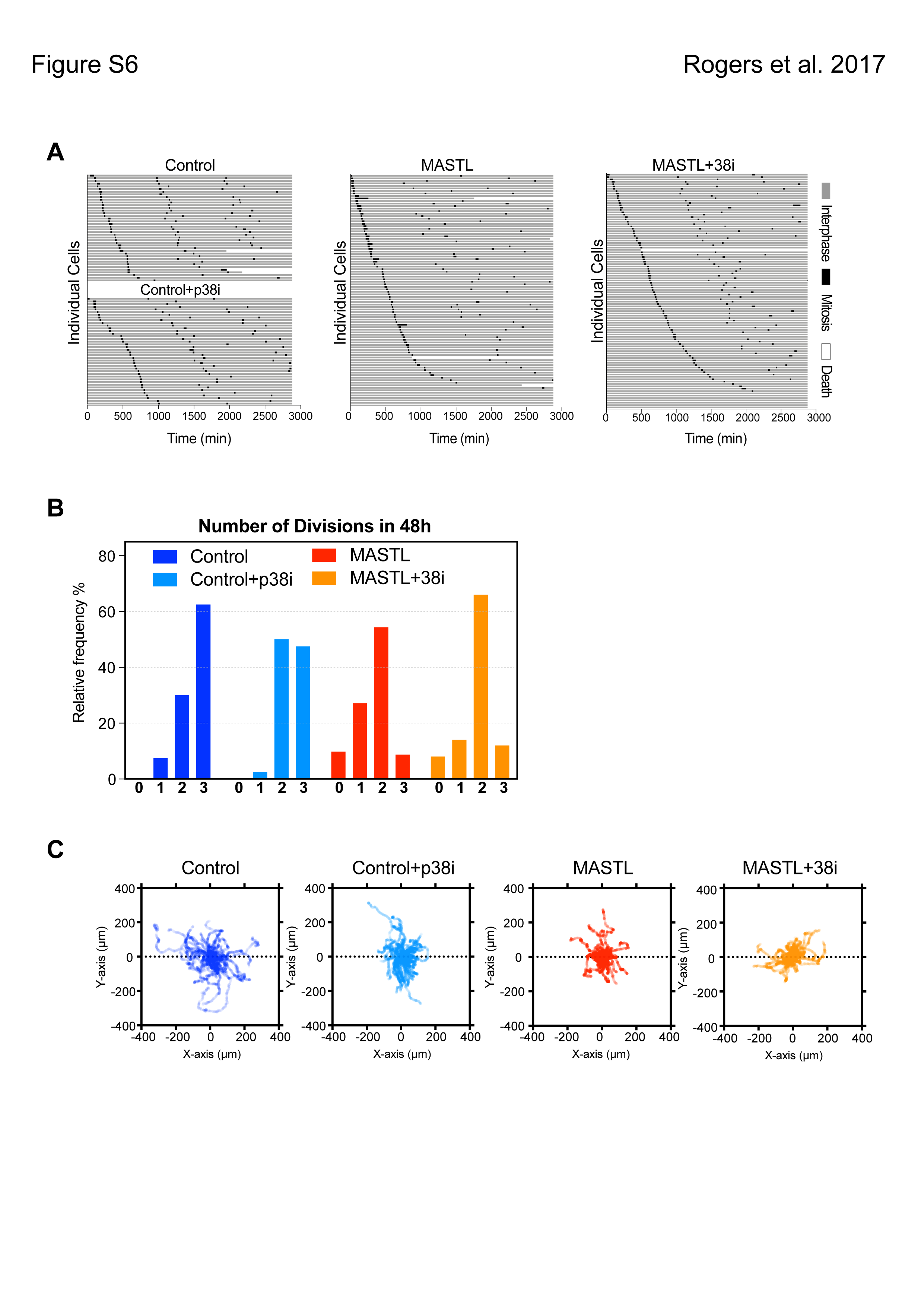

Supplement: Supplementary file 10 — Figure S6 [file 41388_2018_295_MOESM10_ESM.tif]

Figure S7

Rogers et al. 2017

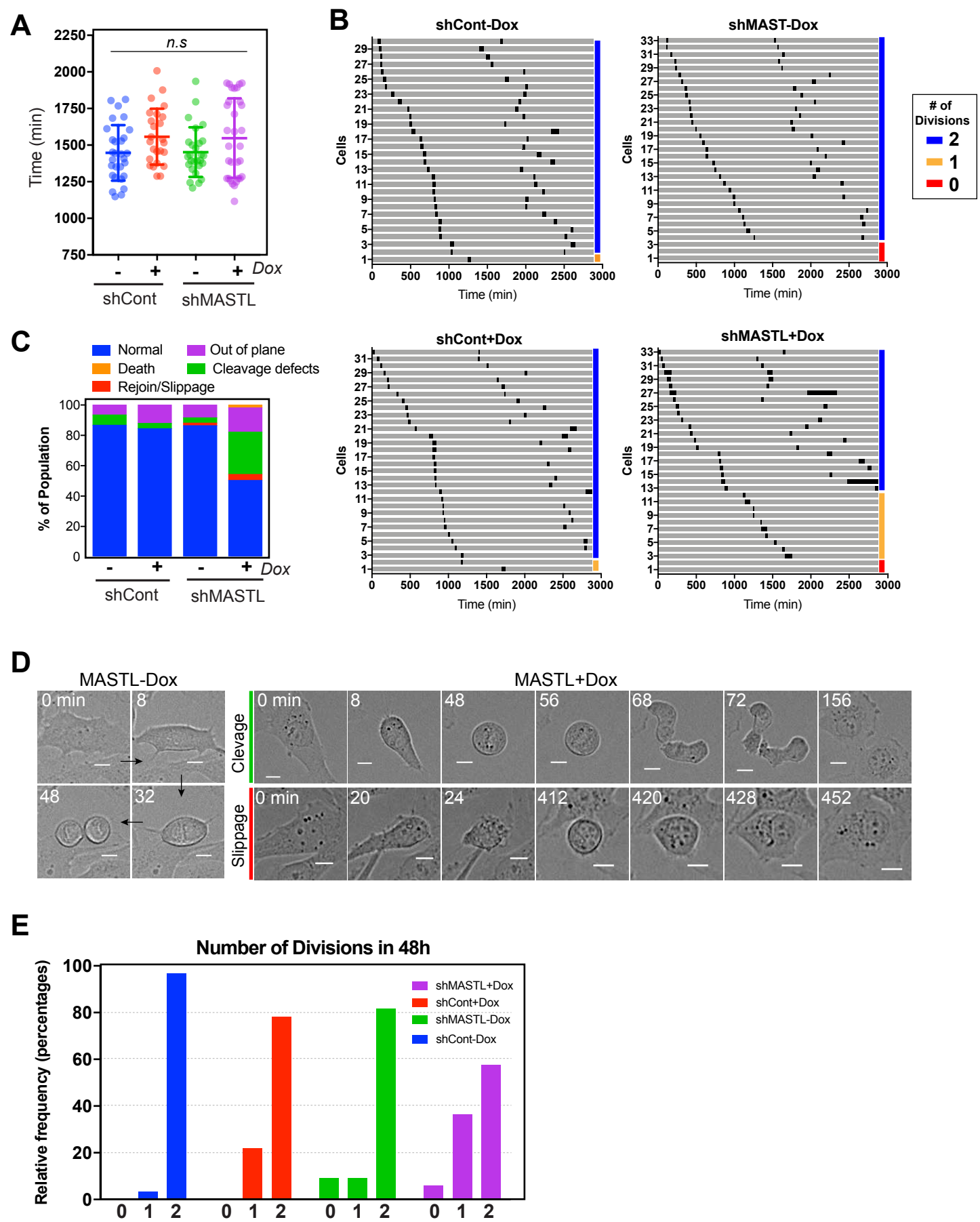

Supplement: Supplementary file 11 — Figure S7 [file 41388_2018_295_MOESM11_ESM.pdf]
